# A prelude to the proximity interaction mapping of CXXC5

**DOI:** 10.1038/s41598-021-97060-6

**Published:** 2021-09-02

**Authors:** Gamze Ayaz, Gizem Turan, Çağla Ece Olgun, Gizem Kars, Burcu Karakaya, Kerim Yavuz, Öykü Deniz Demiralay, Tolga Can, Mesut Muyan, Pelin Yaşar

**Affiliations:** 1 Department of Biological Sciences, Middle East Technical University, 06800 Ankara, Turkey; 2 Department of Computer Engineering Middle, East Technical University, 06800 Ankara, Turkey; 3 Cansyl Laboratories, Middle East Technical University, 06800 Ankara, Turkey; 4 Present Address: Cancer and Stem Cell Epigenetics Section, Laboratory of Cancer Biology and Genetics, Center for Cancer Research, National Cancer Institute, National Institutes of Health, Bethesda, MD 20892 USA; 5 Present Address: Epigenetics and Stem Cell Biology Laboratory, Single Cell Dynamics Group, National Institute of Environmental Health Sciences, Research Triangle Park, NC 27709 USA

**Keywords:** Biochemistry, Cancer, Cell biology, Molecular biology

## Abstract

CXXC5 is a member of the zinc-finger CXXC family proteins that interact with unmodified CpG dinucleotides through a conserved ZF-CXXC domain. CXXC5 is involved in the modulation of gene expressions that lead to alterations in diverse cellular events. However, the underlying mechanism of CXXC5-modulated gene expressions remains unclear. Proteins perform their functions in a network of proteins whose identities and amounts change spatiotemporally in response to various stimuli in a lineage-specific manner. Since CXXC5 lacks an intrinsic transcription regulatory function or enzymatic activity but is a DNA binder, CXXC5 by interacting with proteins could act as a scaffold to establish a chromatin state restrictive or permissive for transcription. To initially address this, we utilized the proximity-dependent biotinylation approach. Proximity interaction partners of CXXC5 include DNA and chromatin modifiers, transcription factors/co-regulators, and RNA processors. Of these, CXXC5 through its CXXC domain interacted with EMD, MAZ, and MeCP2. Furthermore, an interplay between CXXC5 and MeCP2 was critical for a subset of CXXC5 target gene expressions. It appears that CXXC5 may act as a nucleation factor in modulating gene expressions. Providing a prelude for CXXC5 actions, our results could also contribute to a better understanding of CXXC5-mediated cellular processes in physiology and pathophysiology.

## Introduction

The methylation of mammalian genomic DNA, which predominantly arises post-replicatively at the 5’ position of the cytosine base in the context of CpG dinucleotides, varies across cell types, developmental stages, physiological and pathophysiological conditions^1^. Acting as stable and heritable epigenetic marks, methylated CpGs present in 80% of CpGs in the genome and involve both genic and intergenic regions^2^. Methylated CpGs are specifically recognized and bound by methyl-CpG-binding proteins, which in turn generate a chromatin environment refractory to transcription by recruiting a variety of chromatin modifiers^3,4^.

Although the majority of CpGs are methylated, about 70% of annotated human gene promoters are associated with unmethylated DNA stretches called CpG islands (CGIs), which are rich in C and G nucleotides with a high density of CpG dinucleotides^5,6^. The structurally and functionally distinct zinc-finger (ZF)-CXXC family proteins interact with unmodified CpG dinucleotides through a highly conserved ZF-CXXC domain characterized by two consecutive cysteine-rich motifs (CXXCXXC) that associate with two Zn^++^ ions forming zinc-finger structures. Upon binding to unmethylated CpG motifs with varying affinities and specifies within the context of surrounding base composition^7^, the ZF-CXXC family proteins establish a chromatin architecture directly through chromatin-modifying enzymatic activities and/or indirectly through the recruitment of chromatin-modifiers to modulate gene expressions^4,8,9^.

CXXC5, also known as RINF (Retinoid-Inducible Nuclear Factor) and WID (WT1-Induced Inhibitor of Dishevelled), is a member of the ZF-CXXC family. *CXXC5* is located on chromosome 5q31.2 and encodes a 322 amino-acid protein with a predicted molecular mass of 33 kDa^8,10^. Retinoic acid^11^, transforming growth factor-β^12^, bone morphogenetic protein 4^13,14^, Wnt3a^15–17^, and estrogen^18–21^ modulate the expression of *CXXC5* in experimental systems. The encoded CXXC5 protein alters gene expressions^13,17,21–27^ that result in the modulation of diverse cellular events, including signal transduction, DNA damage response, metabolism, proliferation, differentiation, and death^11–13,15,21,22,24,25,27–30^.

However, the underlying mechanism by which CXXC5 regulates gene expressions remains unclear. We recently showed that CXXC5 is an unmethylated CpG binder but lacks an intrinsic transcription regulatory function^21^. This raises the possibility that CXXC5 acts as a nucleation factor to establish a transcription state restrictive or permissive for transcription by interacting with transcription factors, transcription co-regulatory proteins, histone, and/or DNA modifiers. Consistent with this prediction, CXXC5 appears to be involved in epigenetic alterations, including CpG methylations and histone modifications by recruiting TET2 (the ten-eleven translocation methylcytosine dioxygenase 2) at a subset of CGI promoters that results in the modulation of gene expression in plasmacytoid dendritic cells^23^. Similarly, CXXC5 was recently shown to influence the establishment and maintenance of DNA methylation^22^ as well as hydroxymethylation in embryonic stem cells^22^ and immature erythroid cells^27^. It appears that CXXC5 regulates the transcription of *TET1* and *TET2* and interacts with TET1 and TET2 to modulate the expression of pluripotency genes, including Nanog and Oct4 (Octamer-Binding Protein 4)^22^. Nanog and Oct4, in turn, cooperate with CXXC5 as the protein partner at a subset of gene promoters and enhancers to modulate the expression of genes involved in the facilitation of differentiation^22^. Likewise, it was recently reported that the enhanced synthesis and interaction of CXXC5 with TET2 could pose challenges for the treatment of castration-resistant prostate cancer by increasing the accessibility of non-canonical DNA binding sites for androgen receptor^31^. Moreover, the interaction of CXXC5 with TET2 could affect the stability of TET2^32^. It was also reported that CXXC5 inhibits the expression of *CD40L* (CD40 ligand gene) by interacting with histone-lysine N-methyltransferase SUV39H1 (KMT1A) at the gene promoter^26^. Furthermore, CXXC5 was reported to interact with FOXL2 (Forkhead Box L2)^33^, RBPJ (Recombination Signal Binding Protein For Immunoglobulin Kappa J Region)^24^, Sall4 (Spalt Like Transcription Factor 4)^34,35^, members of SMAD (Mothers against decapentaplegic homolog) family^29,36^, and VDR (Vitamin D receptor)^30^ to modulate gene expressions. Additionally, CXXC5 was shown to serve as a negative feedback regulator of the Wnt/β-catenin signaling pathway by interacting with the Dvl (Dishevelled) protein in the cytoplasm of dermal fibroblast^14–16,37^.

Since proteins perform their functions in a network of proteins whose identities and amounts change temporally and spatially in response to intrinsic and extrinsic stimuli in a lineage-specific manner, the identification of protein partners of CXXC5 would critically contribute to a better understanding of the mechanisms of CXXC5-mediated cellular processes in physiology and pathophysiology. Following its introduction, the proximity-dependent biotinylation approach (BioID) has been effectively used for the identification of interacting partners of many proteins^38,39^. BioID is based on the genetic fusion of a mutant *E. coli* biotin ligase enzyme, BirA*(R118G), which is defective in both self-association and DNA binding, to a protein-of-interest to biotinylate proximity proteins^40,41^. Biotinylated proteins are then selectively isolated with biotin-affinity capture and identified with mass spectrometry (MS). Using the BioID-MS approach, we found that CXXC5 interacts with a large number of proteins mainly grouped in the regulation of gene expression which further clustered into proteins involved in DNA, chromatin, and RNA modifications. Of the proteins, we selectively verified that CXXC5 through its CXXC domain interacts with EMD (Emerin), MAZ (MYC Associated Zinc Finger Protein), and MeCP2 (Methyl-CpG Binding Protein 2). We also found that an interplay between CXXC5 and MeCP2 contributes to the expression of some of the CXXC5 target genes. Since CXXC5 lacks an intrinsic transcription regulatory function and enzymatic activity but is an unmethylated CpG binder, our results, together with others, imply that CXXC5 may act as a nucleation factor/molecular scaffold for gene expressions.

## Results

### Expression of the CXXC5-BirA* fusion protein in MCF7 cells

**Figure 1:**
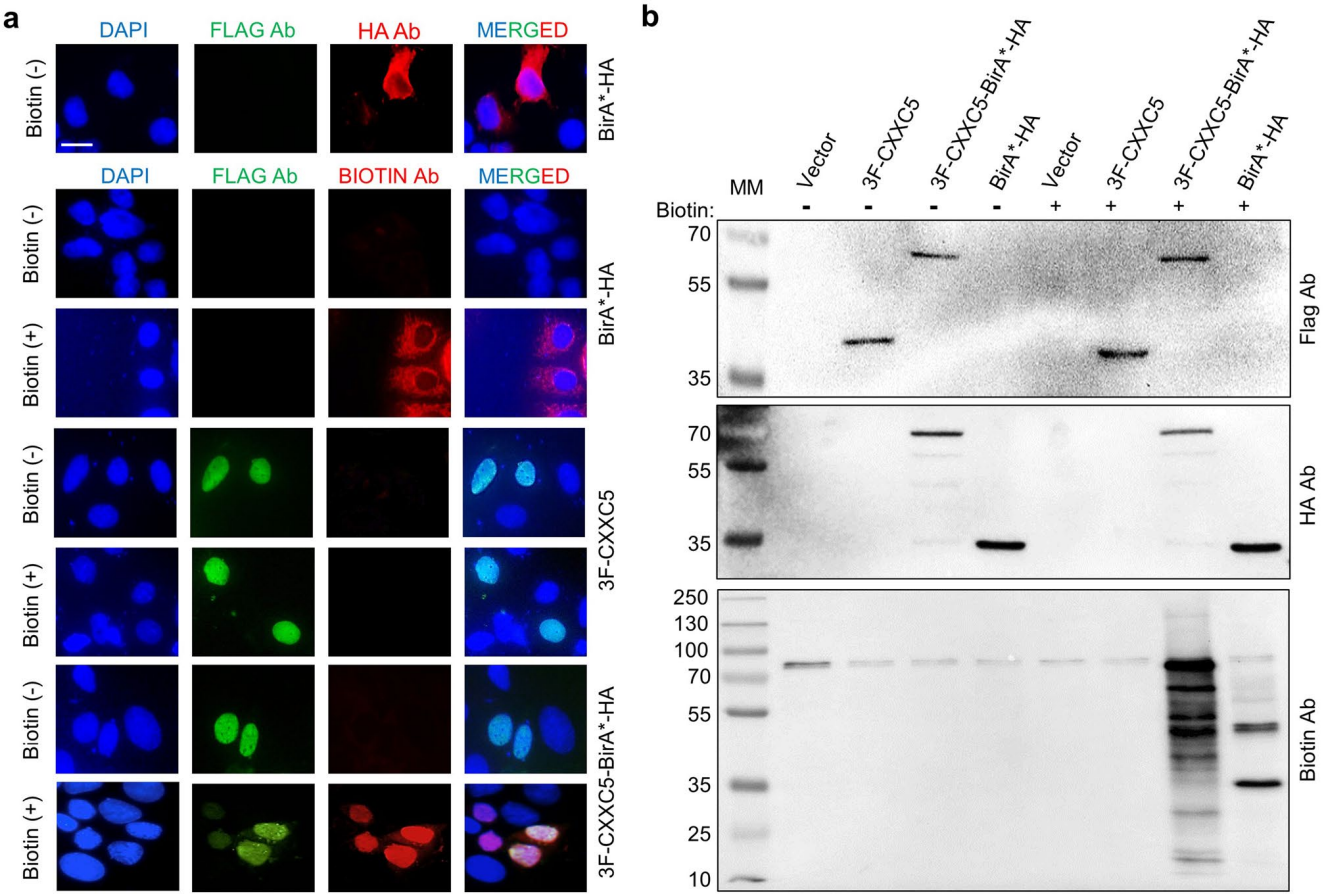
Biotinylation of endogenous proteins in MCF7 cells. MCF7 cells were transiently transfected for 24 h with an expression vector (pcDNA3.1) bearing the cDNA for 3F-CXXC5, 3F-CXXC5-BirA*-HA, or BirA*-HA. Cells were then treated without (-) or with ( +) biotin (50 μM) and ATP (1 mM) for 16 h. (**a**) Transiently transfected cells were subjected to immunocytochemistry using the Flag, the HA, or the Biotin antibody followed by an Alexa Fluor 488 (green fluorescein) conjugated goat anti-mouse IgG for the Flag antibody, or an Alexa Fluor 594 (red fluorescein) conjugated goat anti-rabbit IgG for the HA and the Biotin antibody. The scale bar is 20 µm. (**b**) Total protein extracts of MCF7 cells were subjected to SDS-10%PAGE followed by WB using the Flag, the HA, or the Biotin antibody followed by an HRP-conjugated goat-anti mouse secondary antibody for the Flag (Advansta R-05071–500) or goat-anti-rabbit secondary antibody for the HA or the Biotin antibody (Advansta R-05072–500). Molecular masses (MM) in kDa are indicated.

Although the underlying mechanism(s) is unclear, CXXC5 as a CpG dinucleotide binder is involved in gene expressions. Since the dynamically changing network of protein environment is a critical determinant for proteins to perform their functions in a cell- and signaling pathway-dependent manner, the identification of interacting partners of CXXC5 could provide critical information about the mechanisms of target gene expressions. To begin to address this issue, we utilized the BioID-MS approach. To generate the protein components of BioID, we genetically fused the 3xFlag-CXXC5 (3F-CXXC5) cDNA to the 5’ end of sequences encoding the BirA*-HA cDNA present in the pcDNA expression vector, pcDNA3.1-BirA*(R118G)-HA (BirA*-HA). To ensure that the genetic fusion of 3F-CXXC5 to BirA*-HA does not affect the synthesis, the intracellular localization, and the biotinylation ability of the CXXC5-BirA* fusion protein, we initially carried out immunocytochemistry (ICC) and western blot (WB) analyses in transiently transfected MCF7 cells derived from a breast adenocarcinoma. The expression vector bearing the BirA*-HA, 3F-CXXC5, or 3F-CXXC5-BirA*-HA cDNA was transiently transfected into MCF7 cells for 24 h. Cells were then treated without or with 50 μM biotin and 1 mM ATP for 16 h followed by ICC (Fig. 1a) and WB (Fig. 1b & Supplementary Information Fig. S1) using an antibody specific for the Flag, HA, or biotin. Results revealed that BirA*-HA displaying an expected molecular mass (MM) of 33 kDa was primarily present in the cytoplasm; whereas, 3F-CXXC5 with about 37 kDa MM in the absence or presence of exogenously added biotin was localized in the nucleus, as we showed previously^19^. Similarly, 3F-CXXC5-BirA*-HA with a predicted MM of 70 kDa was localized in the nucleus independently of the exogenously added biotin. Importantly, the detection of many biotinylated proteins only in the presence of biotin in transfected cells synthesizing 3F-CXXC5-BirA*-HA assessed with WB indicates that the fusion protein is functional as well.

### CXXC5 associated proteins in MCF7 cells

Based on the synthesis and intracellular location of the functional 3F-CXXC5-BirA*-HA fusion protein, we then carried out BioID assays in MCF7 cells. Cells were transiently transfected with the expression vector bearing none (EV), the BirA*-HA, or the Flag-CXXC5-BirA*-HA cDNA for 24 h. Cells were then treated in the absence or presence of 50 μM biotin and 1 mM ATP for 16 h. Biotinylated proteins in cell lysates were captured with streptavidin-conjugated magnetic beads. Protein fragments following on-bead tryptic proteolysis of the captured proteins were subjected to mass spectrometry (MS). Subtractive analyses of identified proteins from EV, BirA*-HA, and 3F-CXXC5-BirA*-HA synthesizing cells as two biological replicates with two technical repeats revealed 108 proximal interactors of CXXC5 (Supplementary Information, Table S1). It should be noted that none of the proteins we identified is coincident with reported protein partners of CXXC5 except for TNRC18^42^ which was present in one of the biological replicates of our BioID experiments. Differences in protein profiles are likely due to dynamically changing protein abundances and protein complex compositions in distinct cell types. It is also possible that the genetic fusion of CXXC5 to BirA* altered the native conformational features of the protein, thereby modifying the *in cellula* protein interaction profile of the protein.

Gene ontology analyses for biological functions using the DAVID bioinformatics tool^43^ suggest that CXXC5 interacts with proteins largely grouped in the regulation of gene expression, which can further be sub-grouped into proteins involved in DNA, chromatin, and RNA processes (Supplementary Information, Fig. S2). Proteins identified as transcription factors includes ADNP, AP2A (TFAP2A), BCLAF1, CTCF, CUX1, DIDO1, ELF1, EMSY (C11orf30), GRHL2, LIN54, MAZ, NFIB, NFIX, NR2C2, RFX1, RREB1, SCML2, TRPS1, ZNF148, and ZNF638. Transcription co-regulatory proteins comprise CCAR2, HCFC1, LYRIC, MKL2, SNW1, SP110, TCF20, and TIF1B (TRIM28). DNA and Chromatin modifiers include BAZ1A, CHD4, CHD8, COR1B, HMGX4, KDM2A (CXXC8), KMT2A (CXXC7), KMT2B (CXXC10), GATAD2A, GATAD2B, MeCP2, NSD2, RUVB1, SMCA5, SMHD1, TOP2A, TOP2B, TOX4, and XRCC6. The group of proteins involved in RNA processing, binding, and transport encompasses CPSF6, DDX21, DDX5, DHX9, DKC1, HNRPR, NAT10, NONO, NUCL (NCL), PAIRB, ROA2, SF3B2, SRRM1, TADBP, and THOC4. Also, the proximity interaction partners of CXXC5 include architectural proteins Emerin (EMD) as well as LAP2α and LAP2β, both of which are splice variants encoded by *TMPO*.

### Interaction of CXXC5 with EMD and MAZ

The initial validation of interactions between the putative interactors with the endogenous CXXC5 by the use of immunoprecipitation in MCF7 cells proved to be difficult. This was due to low levels of endogenous CXXC5 synthesis and the efficiency of the available antibodies from different resources for the immunoprecipitation of CXXC5 (Supplementary Information, Fig. S3). To circumvent these problems, we used the Human Embryonic Kidney 293 (HEK293) cells that exhibit high transfection efficiency^44^. Initial screening of some of the CXXC5 proximity interacting partners identified with BioID by the use of transient transfections followed by co-immunoprecipitation (Co-IP) in HEK293 cells revealed that CXXC5 could interact, for example, with EMD, MAZ, and MeCP2 proteins but not with LAP2α (Thymopoietin; Lamina-Associated Polypeptide 2, Isoform alpha), RUVBL1 (RuvB Like AAA ATPase 1) or SNW1 (SNW Domain Containing 1) protein (Supplementary Information, Fig. S4). Based on these observations, we selected EMD, MAZ, and MeCP2 to verify that they are indeed interacting protein partners of CXXC5.

**Figure 2:**
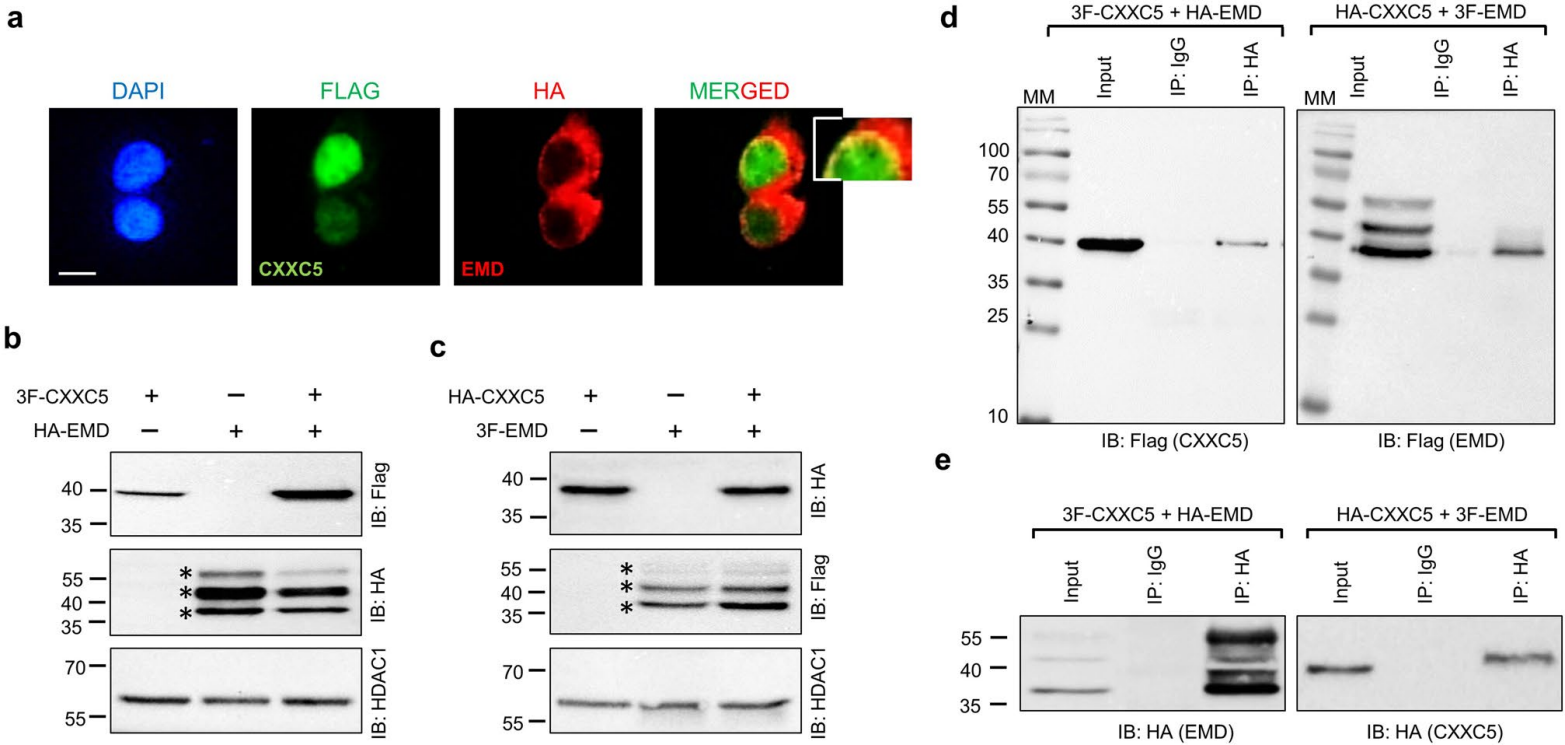
Interaction of EMD and CXXC5. (**a**) To assess the intracellular localization of EMD and CXXC5 when co-synthesized, HEK293 cells were transiently transfected for 48 h with the expression vector bearing 3F-CXXC5 and HA-EMD cDNA. Cells were then subjected to ICC using the Flag (green channel) or the HA (red channel) antibody. DAPI was used for DNA staining. The scale bar is 10 μm. Inset indicates a section with higher magnification. **(b,c)** To examine the protein synthesis, HEK293 cells were transfected with the expression vector bearing **(b)** 3F-CXXC5 and/or HA-EMD cDNA; or (**c**) HA-CXXC5 and/or 3F-EMD cDNA for 48 h. The synthesis of proteins was assessed by WB using the HA or the Flag antibody. HDAC1 used as a loading control was probed with the HDAC1 antibody. Star denotes distinct EMD species (**d,e**)**.** The nuclear extracts (500 μg) of transiently co-transfected HEK293 cells were subjected to Co-IP with the HA (**d**) or the isotype-matched IgG. 50 μg of nuclear lysates was used as input control. The precipitates were subjected to SDS-10%PAGE followed with WB using analyzed using the Flag **(d)** or the HA **(e)** antibody. Molecular masses (MM) in kDa are indicated.

EMD, a member of the nuclear lamina-associated protein family^45^, is a serine-rich inner nuclear membrane protein with a predicted molecular mass (MM) of 28.9 kDa and is involved in the organization of chromatin structure, nuclear assembly, and gene expressions^46^. To validate the interaction between CXXC5 and EMD, we transiently transfected HEK293 cells with the expression vector bearing 3F-CXXC5 and/or HA-EMD cDNA for 48 h (Fig. 2). We also transiently transfected cells with expression vectors bearing cDNAs with converse tag sequences: 3F-EMD and/or HA-CXXC5 cDNA to ensure that the nature of tags does not alter the intracellular localization and/or interactions of the putative protein partners (data not shown). 3F-CXXC5 is primarily localized to the nucleus, whereas HA-EMD shows a nuclear membrane/periphery staining that partially overlaps with the staining of 3F-CXXC5 as well (Fig. 2a & Inset). HA-EMD or 3F-EMD, in transfected cells, shows three distinct protein species with discrete electrophoretic migration ranging from 35 to 55 kDa, in contrast to 3F-CXXC5 or HA-CXXC5, both of which display a single electrophoretic species with an MM of about 37 kDa (Fig. 2b,c). Although protein species of EMD were not studied further, they likely represent isoforms with differentially processed post-translational modifications^47–49^. Immunoprecipitation of nuclear extracts of transiently transfected cells that co-synthesize 3F-CXXC5 and HA-EMD using the HA antibody (Fig. 2d,e; Supplementary Information, Fig. S5) together with protein A and G magnetic beads followed by immunoblotting with the Flag (Fig. 2d) or the HA (Fig. 2e) antibody indicates the presence of both EMD and CXXC5 in the immunoprecipitants. This demonstrates that CXXC5 and EMD interact. Interestingly, CXXC5 displayed an interaction only with the 35 kDa EMD species (Fig. 2d,e).

**Figure 3:**
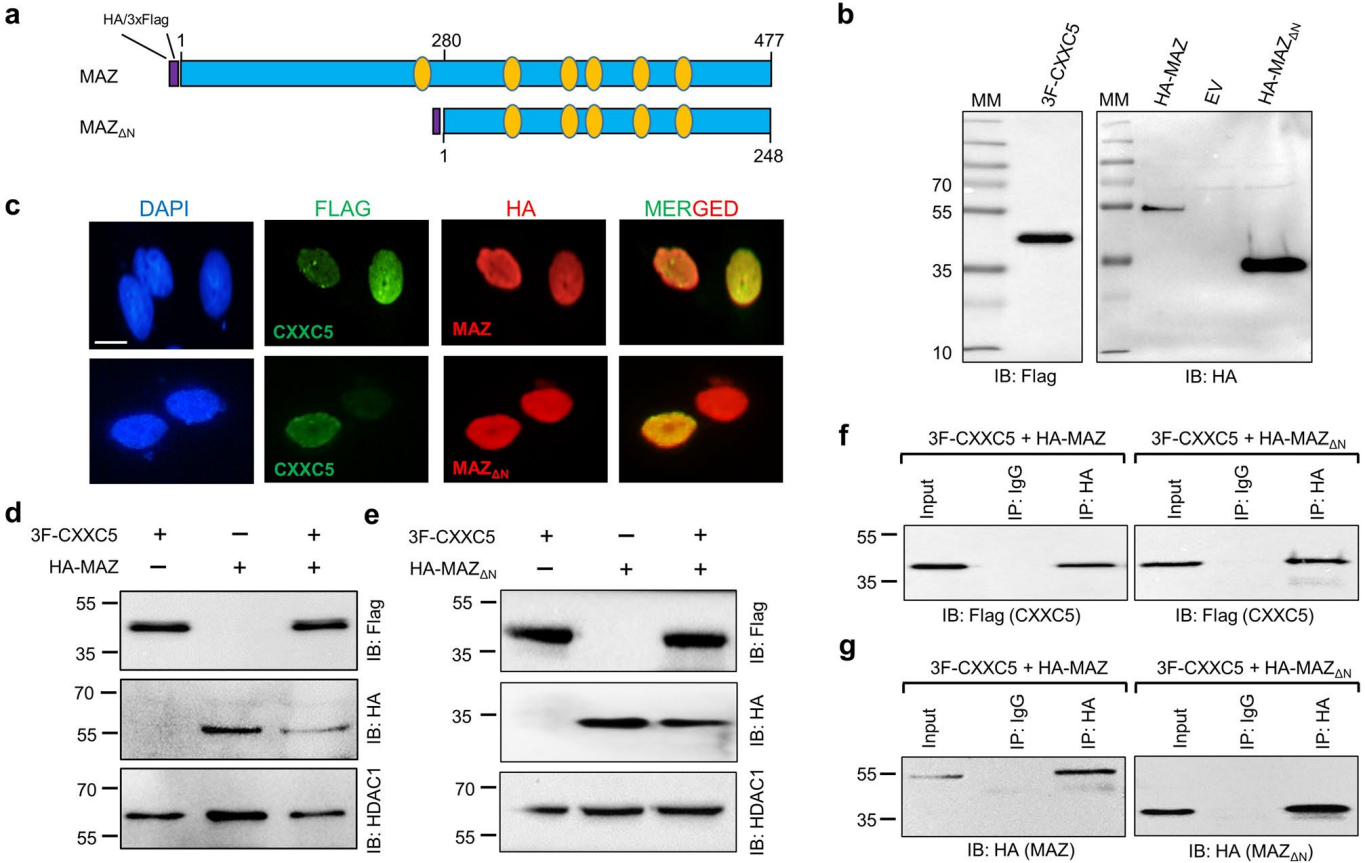
Interaction of MAZ and CXXC5. (**a**) Schematics of the full length (MAZ) or the amino-terminally truncated MAZ (MAZ_ΔN_) variant bearing a Flag or HA tag at the amino-terminus. Ovals in orange color indicate the C2H2-type zinc finger domains. **(b)** To evaluate the protein synthesis, HEK293 cells were transfected with the expression vector bearing none (EV), the HA-MAZ, HA-MAZ_ΔN,_ or 3F-CXXC5 cDNA. Nuclear extracts were subjected to SDS-10%PAGE followed by WB using the HA or the Flag antibody. **(c)** To assess the intracellular localization of the proteins, HEK293 cells were transiently transfected for 48 h with the expression vector bearing the 3F-CXXC5 and the HA-MAZ or HA-MAZ_ΔN_ cDNA. Cells were then subjected to ICC using the Flag (green channel) or the HA (red channel) antibody. DAPI staining indicates the nucleus. The scale bar is 10 μm. **(d,e)** To assess the co-synthesis of proteins, the nuclear extracts of HEK293 cells, transiently transfected with the expression vector bearing 3F-CXXC5 and/or (**d**) the HA-MAZ or (**e**) the HA-MAZ_ΔN_ cDNA, were subjected to WB analyses. Proteins were immunoblotted (IB) with the Flag or HA-antibody. HDAC1 used as a loading control was probed with the HDAC1 antibody. **(f,g)** The nuclear extracts, 500 µg, of transiently co-transfected HEK293 cells were subjected to Co-IP with the HA or the isotype-matched IgG. 10% of nuclear lysate was used as input control. The precipitates were subjected to SDS-10%PAGE followed with WB using the Flag (**f**) or the HA (**g**) antibody. Molecular masses (MM) in kDa are indicated.

MAZ is a transcription factor with C2H2-type zinc finger motifs that can bind GC-rich promoters of target genes to control transcriptional processes^50^. We had carried out the initial screening of the CXXC5 interaction with MAZ using a MAZ cDNA that encodes an amino-terminally truncated variant with an estimated MM of 28 kDa (MAZ_ΔN_), whereas the full-length MAZ is about 51 kDa (Fig. 3a). We observed in transiently transfected HEK293 cells that HA-MAZ and HA-MAZ_ΔN_ display electrophoretic mobility of 55 and 33 kDa, respectively (Fig. 3b). In transiently co-transfected HEK293 cells, the nuclearly localized (Fig. 3c) and co-synthesized 3F-CXXC5 and HA-MAZ (Fig. 3d) or HA-MAZ_ΔN_ (Fig. 3e) showed interactions, as 3F-CXXC5 was immunoprecipitated with the HA antibody (Fig. 3f) in the co-presence of HA-MAZ or HA-MAZ_ΔN_ (Fig. 3g). These results indicate that MAZ, through the carboxyl-terminus, interacts with CXXC5.

### Interaction of CXXC5 with MeCP2

As a member of the methyl-CpG binding protein family (MBP) with a conserved methyl-cytosine binding domain (MBD), MeCP2 with a predicted MM of 53 kDa binds to methylated CpG dinucleotides and unmethylated DNA^3^ in contrast to CXXC5 which preferentially interacts with unmethylated CpG dinucleotide containing DNA^7,21^. The binding of MBPs to DNA as DNA methylation readers alters chromatin structure by recruiting chromatin remodelers and histone modifiers to modulate gene expressions^3^.

**Figure 4:**
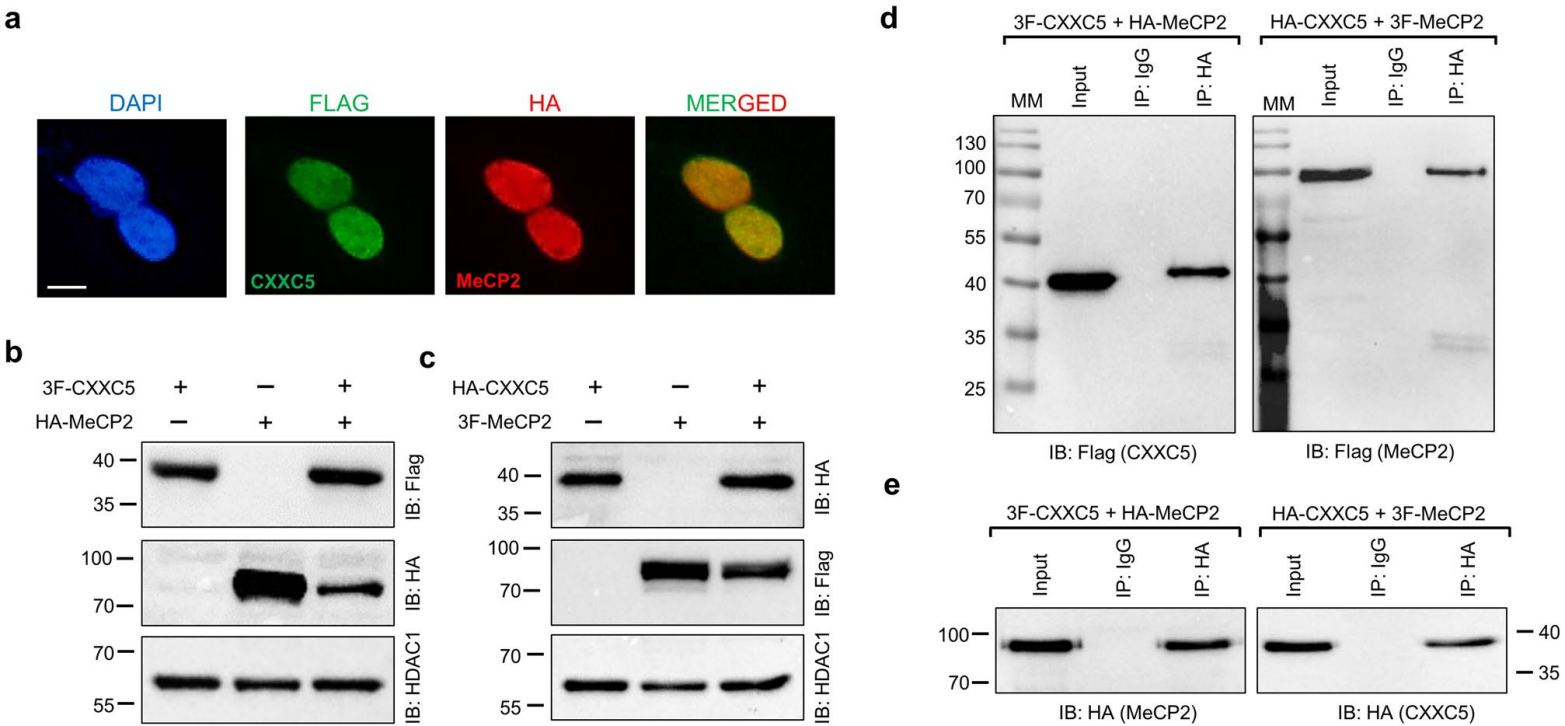
Interaction of CXXC5 and MeCP2. (**a**) The intracellular localization of CXXC5 and MeCP2 when co-synthesized is assessed with ICC of HEK293 cells transiently transfected for 48 h with the expression vector bearing the 3F-CXXC5 or the HA-MeCP2 cDNA. The Flag (green channel) or the HA (red channel) antibody was used to detect 3F-CXXC5 and HA-MeCP2, respectively. DAPI was used for DNA staining. The scale bar is 10 μm. (**b,c**) To examine the co-synthesis of CXXC5 and MeCP2, nuclear extracts of HEK293 cells transiently transfected with the expression vector bearing (**b**) the 3F-CXXC5 and/or the HA-MeCP2 or **(c)** HA-CXXC5 and/or the 3F-MeCP2 cDNA were subjected to WB analysis. Proteins were immunoblotted (IB) with the Flag or HA antibody. HDAC1 used as a loading control was probed with the HDAC1 antibody. (**d,e**) The nuclear extracts of HEK293 cells co-synthesizing 3F-CXXC5 and HA-MeCP2, or co-synthesizing HA-CXXC5 and 3F-MeCP2 were subjected to Co-IP using the HA antibody or the isotype-matched IgG followed by immunoblotting using (**d)** the Flag or (**e**) the HA antibody. Molecular masses (MM) in kDa are indicated.

To examine the interaction of CXXC5 with MeCP2, HEK293 cells were transiently transfected with an expression vector bearing the 3F-CXXC5, or HA-CXXC5, and/or HA-MeCP2, or 3F-MeCP2, cDNA for 48 h. In HEK293 cells, HA-MeCP2, as 3F-CXXC5, localizes to the nucleus (Fig. 4a). Immunoblotting of nuclear extracts of transiently transfected HEK293 cells revealed that HA-MeCP2 or 3F-MeCP2 primarily displays electrophoretic mobility of about 80 kDa, as shown previously^51^, when synthesized alone or together with 3F-CXXC5 or HA-CXXC5 (Fig. 4b,c). The apparent higher MM than the estimated MM of MeCP2 is likely due to different post-translational modifications of the protein^52^. Immunoprecipitation of 3F-CXXC5 or 3F-MeCP2 with the HA antibody (Fig. 4d) in the presence of HA-MeCP2 or HA-CXXC5 in immunoprecipitants, respectively, (Fig. 4e) suggests that CXXC5 and MeCP2 are interacting partners.

**Figure 5:**
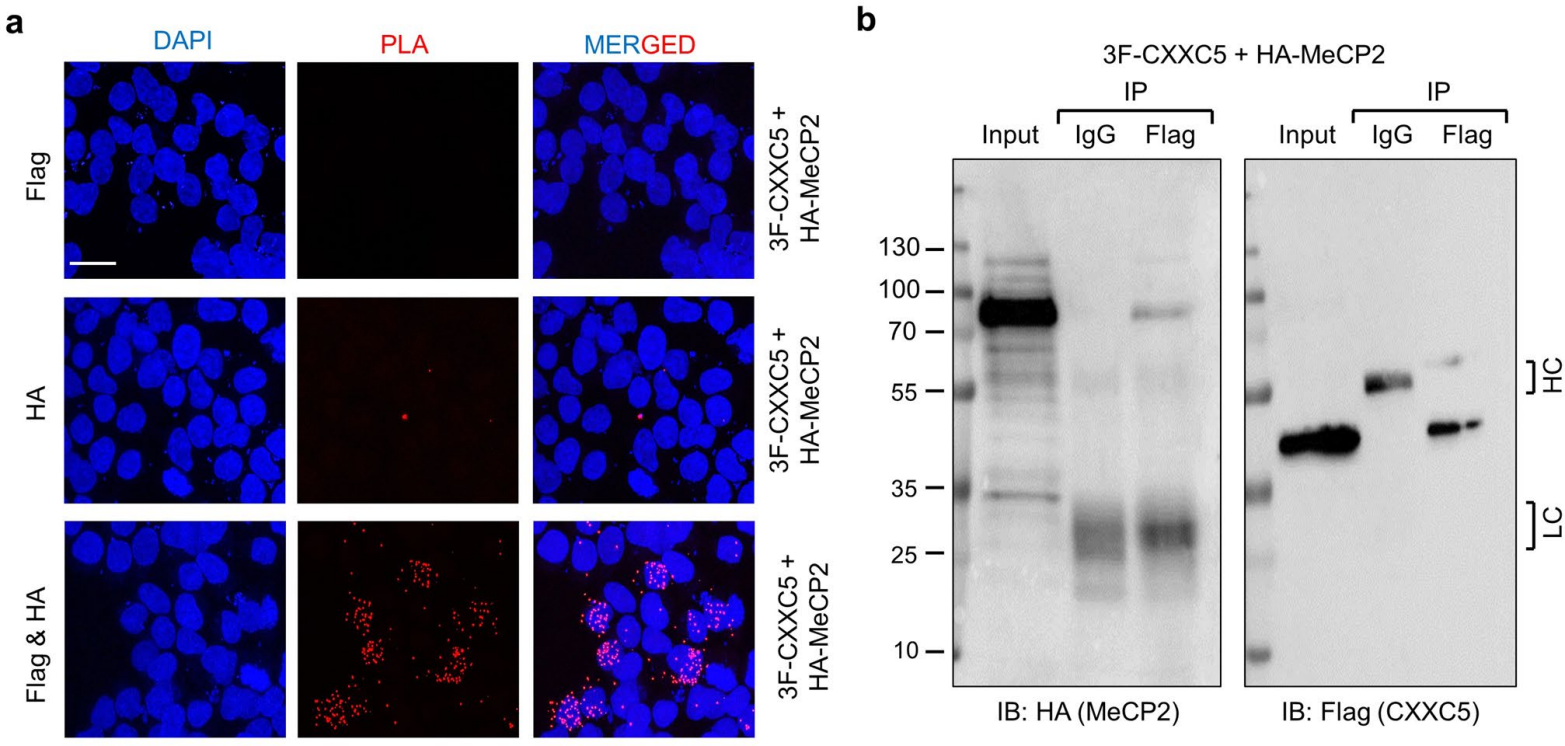
*In cellula* interaction of CXXC5 and MeCP2. (**a**) Proximity ligation assay (PLA). To assess the *in cellula* interaction of CXXC5 and MeCP2, HEK293 cells grown in coverslips were transiently co-transfected the expression vector bearing the 3F-CXXC5 or HA-MeCP2 cDNA. Cells were fixed, permeabilized, blocked, and probed with the HA and/or the Flag antibody. Cells were then subjected to fluorescent probes for circular DNA amplification. DAPI was used for nuclear staining. The scale bar is 25 µm. (**b**) Chromatin immunoprecipitation assay (ChIP)-WB. Co-transfected cells were also subjected to ChIP using the Flag antibody or the isotype-matched IgG followed by immunoblotting using the HA antibody. The membrane was also re-probed with the Flag antibody. HC and LC indicate the heavy and light chain of IgG. 10% of ChIP was used as input control.

The interaction between the unmethylated CpG dinucleotide binder CXXC5 and the DNA methylation reader and histone modifier MeCP2 is perplexing and enticed us to further explore the feature of this interaction. To extend the verification that CXXC5 and MeCP2 are interacting partners, we carried out the proximity ligation assay (PLA). PLA used here utilizes species-specific secondary antibodies conjugated with distinct DNA primers. A hybridization step followed by circular DNA amplification with fluorescent probes to the conjugated DNA primers allows the visualization of proximity spots by fluorescence microscopy^53^. In transiently transfected HEK293 cells synthesizing HA-MeCP2 and/or 3F-CXXC5, the HA or the Flag antibody alone showed virtually no fluorescence signal in cells whereas prominent nuclear fluorescence signals were detectable when cells were probed with both antibodies (Fig. 5a, Supplementary Information Fig. S6). This observation reinforces the conclusion that CXXC5 and MeCP2 are interacting partners.

Moreover, ChIP of cell extracts from HEK293 cells transiently transfected with 3F-CXXC5 and HA-MeCP2 with the Flag antibody followed by immunoblotting using the HA antibody and re-probing with the Flag antibody suggests that CXXC5 and MeCP2 are co-present on chromatin as well (Fig. 5b).

#### Assessing sub-regions critical for CXXC5-MeCP2 interactions

**Figure 6:**
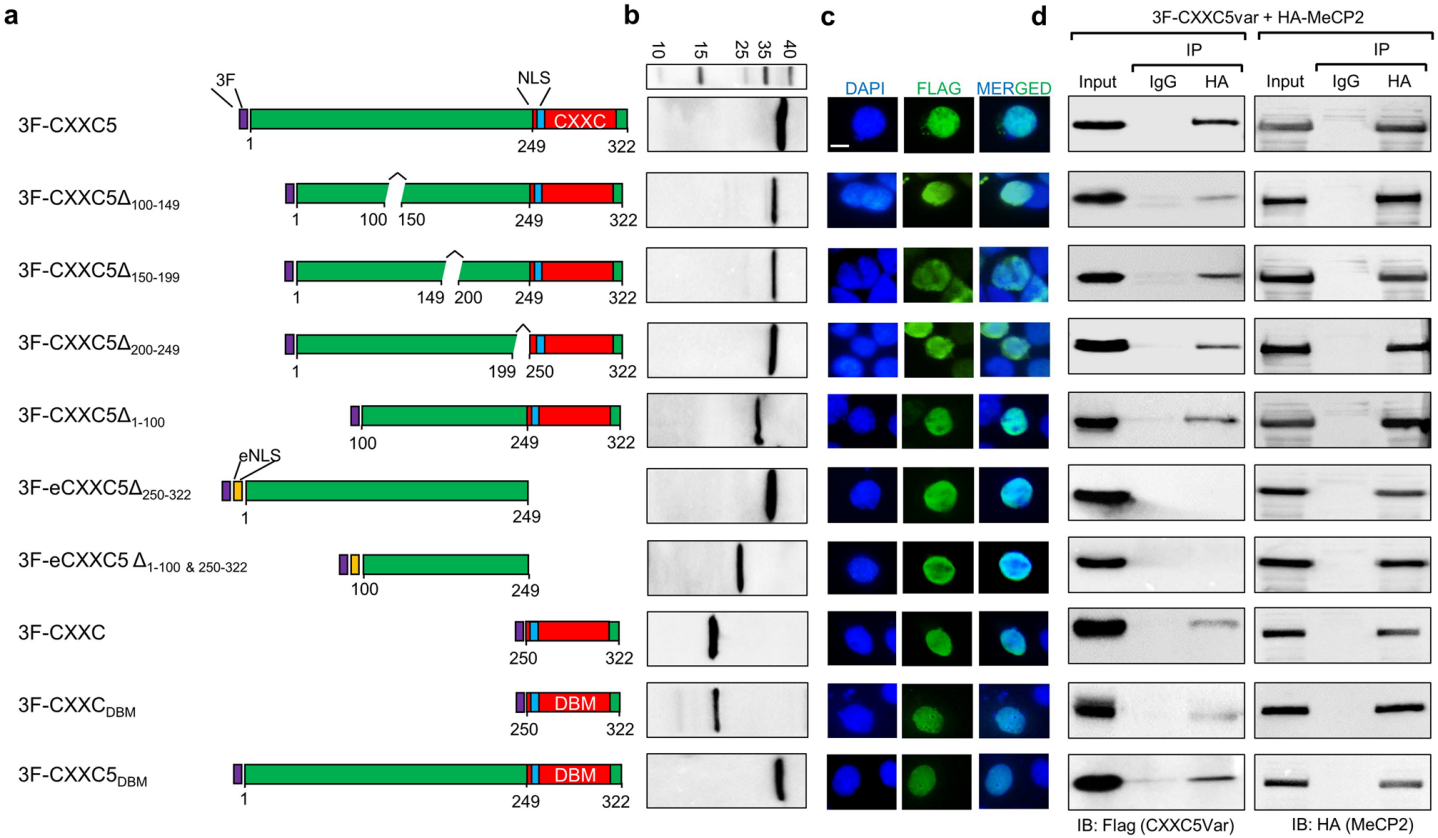
Identification of interaction region(s) of CXXC5 with MeCP2. (**a**) Schematics of CXXC5 variants. 3F denotes 3XFlag tag and eNLS is an NLS signal derived from the SV40 T antigen. DBM denotes DNA binding mutant. (**b**) The synthesis of proteins in transiently transfected HEK293 cells was verified by WB using the Flag antibody. Molecular masses (MM) in kDa are indicated. (**c**) Transiently transfected HEK293 cells were subjected to ICC using the Flag antibody followed by Alexa Fluor 488 conjugated secondary antibody for visualization with a fluorescence microscope. DAPI was used for the nuclei staining. (**d**) HEK293 cells were transiently co-transfected with the expression vector bearing cDNA for a 3F-CXXC5 variant and HA-MeCP2. Nuclear extracts (500 ug) were subjected to Co-IP with the HA antibody or the isotype-matched IgG. The precipitates were subjected to SDS-15%PAGE followed by WB using the Flag antibody or the HA antibody. 10% of nuclear extracts was used as input control.

Based on these results, we wanted to explore a sub-region(s) of CXXC5 critical for the interaction with MeCP2. We generated cDNAs encoding amino-terminally, carboxyl-terminally, or internally truncated CXXC5 proteins. Since CXXC5 localizes to the nucleus through a nuclear localization signal present at the immediate amino-terminus of the CXXC domain^28^, to ensure that truncated CXXC5 variants lacking the CXXC domain also localize to the nucleus by inserting an exogenous nuclear localization signal (eNLS) derived from the SV40 T antigen^54^ between the Flag epitope and a truncated CXXC5 variant, namely 3F-eCXXC5Δ_250-322_ and 3F-eCXXC5Δ_1-100&250-322_ (Fig. 6a). To examine the synthesis and intracellular location of CXXC5 variants, we performed WB from and ICC in transiently transfected HEK293 cells with the use of the Flag antibody. Results revealed that CXXC5 variants were synthesized at expected MMs (Fig. 6b) and were localized primarily to the nucleus (Fig. 6c). To assess a sub-region(s) of CXXC5 critical for MeCP2 interaction, HEK293 cells transiently co-transfected with the expression vector bearing a 3F-CXXC5 variant and the HA-MeCP2 cDNA for 48 h were subjected to Co-IP. The nuclear extracts were immunoprecipitated with the HA antibody and immunoblotted with the Flag antibody followed by re-probing with the HA antibody (Fig. 6d). Results showed that 3F-eCXXC5Δ_250-322_ with the deleted CXXC domain was not detectable in the precipitants. Similarly, 3F-eCXXC5Δ_1-100 & 250–322_ lacking the first 100 amino acids at the amino-terminus together with the deleted CXXC domain did not show an interaction in either immunoprecipitants or in cells as assessed with PLA (Supplementary Information Fig. S6). On the other hand, the remaining of the CXXC5 variants, including the CXXC domain alone, 3F-CXXC, were detectable with the Flag antibody in the HA, but not in the IgG, precipitated lysate. The sequential residues at the domain (threonine, glycine, histidine, and glutamine amino acids, TGHQ) are critical for the binding of the CXXC domain to DNA^23,32,55^. To assess whether DNA binding defective CXXC5 interacts with MeCP2, we generated full-length 3F-CXXC5_DBM_ and 3F-CXXC_DBM_ mutants by converting the TGHQ sequence to AAAA. 3F-CXXC_DBM_ as the full-length 3F-CXXC5_DBM_ retained the interaction with MeCP2. These results collectively suggest that the carboxyl-terminal CXXC domain of CXXC5 is the required region to interact with MeCP2 independently of its ability of binding to DNA.

It should be noted that although the CXXC domain is the required region of CXXC5 for protein interactions, the central region of CXXC5, aa 101–150, could contribute to the stability/affinity of protein interactions. We observed that the 3F-CXXC5Δ_100-149_ variant, similar to the CXXC domain alone (3F-CXXC), consistently showed a lesser degree of interaction with MeCP2 compared to, for example, 3F-CXXC5, 3F-CXXC5Δ_150-199_, 3F-CXXC5Δ_200-249_, or 3F-CXXC5Δ_1-100_.

**Figure 7:**
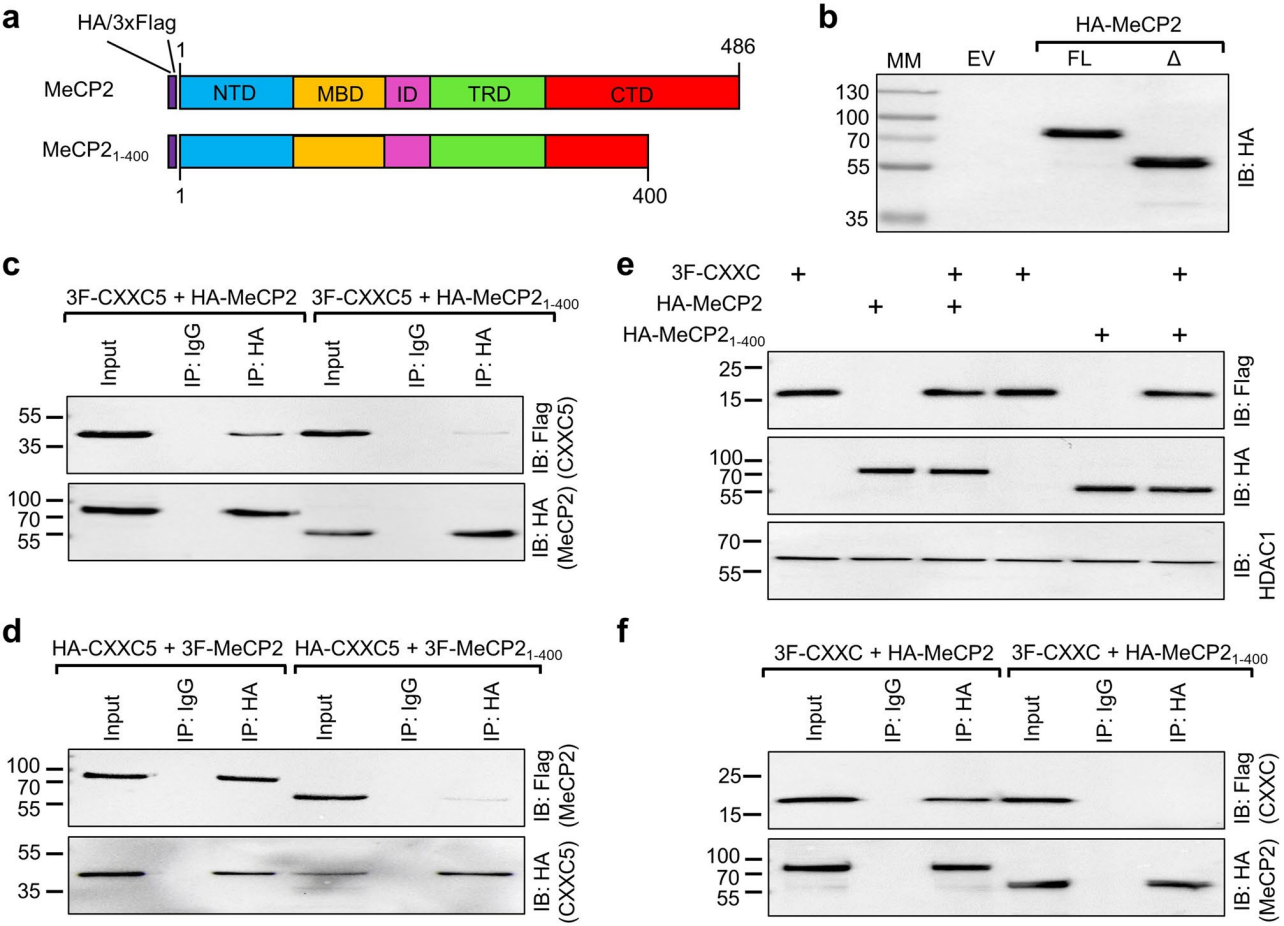
Identification of a sub-region of MeCP2 critical for interacting with CXXC5. (**a**) Schematics of the wild-type MeCP2 and the carboxyl-terminally truncated MeCP2_1-400_ bearing the 3xFlag (3F) or the HA (HA) tag at the amino-terminus. The N-terminal domain (NTD), methyl CpG binding domain (MBD), inter-domain (ID), transcription repression domain (TRD), and C-terminal domain (CTD) containing a WW protein interaction domain are indicated. (**b**) The synthesis of MeCP2 (FL) or HA-MeCP2_1-400_ (Δ) in transiently transfected cells was assessed with WB using the HA antibody. EV indicates empty vector as control and MM denotes molecular masses in kDa. (**c,d**) HEK293 cells were transiently co-transfected with the expression vector bearing cDNA for 3F-CXXC5 and HA-MeCP2 or HA-MeCP2_1-400_. Nuclear extracts were subjected to Co-IP with the HA or the isotype-matched IgG. The precipitates were subjected to WB using the Flag antibody. The membrane was re-probed with the HA antibody. 10% of nuclear extracts was used as input control. Molecular masses (MM) in kDa are indicated. (**e**) Nuclear extracts of HEK293 cells transiently co-transfected with an expression vector bearing cDNA for the 3F-CXXC domain (3F-CXXC), HA-MeCP2, or HA-MeCP2_1-400_, were subjected to WB using the Flag, HA or HDAC1 antibody. Molecular masses (MM) in kDa are indicated. (**f**) Nuclear extracts, 500 µg, co-synthesizing CXXC_,_ and HA-MeCP2, or HA-MeCP2_1-400_, were subjected to Co-IP with the HA antibody or the isotype-matched IgG. The precipitates were subjected to WB using the Flag antibody. The membrane was also re-probed with the HA antibody. 10% of nuclear extracts was used as input control. Molecular masses (MM) in kDa are indicated.

To examine whether we could also locate a region of MeCP2 involved in the interaction with CXXC5, we generated MeCP2 variants. An initial screening of MeCP2 variants suggested that the carboxyl-terminus of MeCP2 is involved in the interaction with CXXC5. A previous detailed study indicated that the carboxyl-terminus of MeCP2 is required for the interaction with FBP11, the protein product of *PRPF40A* (Pre-mRNA Processing Factor 40 Homolog A) and HYPC, the protein product of *PRPF40B* (Pre-mRNA Processing Factor 40 Homolog B)^56^; as the truncation of the carboxyl-terminal 86 amino acids containing the WW protein interaction domain of MeCP2, which also results from a frameshift mutation present in a group of Rett syndrome patients, abrogates interactions with FBP11 and HYPC. In transiently transfected HEK293 cells, the nuclearly localized (data not shown) the HA, or the Flag (data not shown), tagged MeCP2 variant lacking 86 amino acids from the carboxyl terminus (MeCP2_1-400_) (Fig. 7a) displays a MM of about 55 KDa (Δ) compared to the full-length (FL) HA-MeCP2 which exhibits an apparent MM of 80 kDa (Fig. 7b). Immunoprecipitation of HA-MeCP2_1-400_, or Flag-MeCP2_1-400_, with the HA antibody from nuclear extracts of HEK293 cells co-synthesizing 3F-CXXC5, or HA-CXXC5, revealed that the interaction of CXXC5 with MeCP2_1-400_ decreases dramatically compared to HA-MeCP2 or Flag-MeCP2 (Fig. 7c,d; Supplementary Information Fig. S7c,d). These results suggest that the carboxyl-terminus of MeCP2 is a critical region for interaction with CXXC5 as well. Moreover, the CXXC domain of CXXC5, 3F-CXXC, appears to be sufficient for the interaction of CXXC5 with HA-MeCP2 but not HA-MeCP2_1-400,_ as the HA antibody specifically immunoprecipitated 3F-CXXC from nuclear extracts of HEK293 cells co-synthesizing HA-MeCP2 (Fig. 7e,f; Supplementary Information Fig. S7e,f).

Similarly, the immunoprecipitation of 3F-CXXC with HA antibody from cellular extracts of HEK293 cells co-synthesizing HA-EMD or HA-MAZ (Supplementary Information Fig. S8a,b) further suggests that the CXXC domain of CXXC5 is also a critical region for the interaction with EMD or MAZ.

These observations collectively suggest that the CXXC domain as the DNA binding module of CXXC5 also participates in protein interactions, supporting previous findings that CXXC5, through the CXXC domain, interacts with Dvl1^57^, HDAC1 (Histone deacetylase 1)^12^, and functionally associates with ATM (ATM Serine/Threonine Kinase)^28^.

#### Assessing the possible interplay between CXXC5 and MeCP2 in gene expressions

Our findings that CXXC5 and MeCP2 are co-present on chromatin imply an interplay between these proteins that could modulate gene expressions. We previously reported that CXXC5 is involved in the expression of *HDAC11* (Histone deacetylase 11), *NFKBIZ* (NF-kappa-B inhibitor zeta), or *IL12A* (Interleukin-12 subunit alpha) in MCF7 cells^21^ as similarly reported for the *IL12A* gene in plasmacytoid dendritic cells^23^. To explore whether the alterations in the extent of gene expression is due to the engagement of CXXC5 with the promoter region of *HDAC11*, *NFKBIZ,* or *IL12A*, we initially carried out bioinformatics analyses using the Eukaryotic Promoter Database^58,59^, which contains resources of eukaryotic RNA polymerase II promoters with experimentally defined transcription start sites, to locate the promoter region of *HDAC11, NFKBIZ or IL12A*. Results indicate that the promoter of *HDAC11 or NFKBIZ*, as reported previously^60,61^, or *IL12A* is located within a CpG island (Supplementary Information Fig. S9). We also performed bioinformatics analyses using the Cistrome Data Browser which utilizes publicly available ChIP-chip and ChIP-seq datasets for genome-wide locations of transcription factor binding from various biological resources^62,63^, to assess whether CXXC5 is enriched at the promoter region of *HDAC11, NFKBIZ, or IL12A*. We found no CXXC5 dataset generated with the use of human resources at the Cistrome Data Browser. However, our analysis of a recent ChIP-Seq dataset carried out with the use of mouse embryonic stem cells^22^ suggests, at least in one of two replicates of ChIP-seq results, that CXXC5 may interact with the promoter region of *IL12A.* Although there is no MeCP2 ChIP-Seq dataset generated with the use of MCF7 cells, analyses with datasets from IMR-90, a human lung fibroblast cell line, and HCT-166 cells derived from human colon carcinoma revealed that MeCP2 could associate with the promoter region of *HDAC11*, *NFKBIZ*, or *IL12A* (Supplementary Information Fig. S9).

Based on these analyses, we assessed the possible association of CXXC5 or MeCP2 with the promoter region of *HDAC11*, *NFKBIZ*, or *IL12A* in MCF7 cells. We also used as a negative control Exon10 of *CXXC5*, which is highly methylated^64^ and to which MeCP2 does not show binding as assessed with the Cistrome Data Browser (data not shown). Cells transiently transfected with the expression vector bearing the 3F-CXXC5 or HA-MeCP2 cDNA for 48 h were subjected to ChIP using the Flag or HA antibody followed by qPCR using primers specific to the promoter region of *HDAC11*, *NFKBIZ, IL12A*, or Exon10 of *CXXC5*. ChIP-qPCR results indicated that CXXC5 or MeCP2 associates with the promoter region of *HDAC11*, *NFKBIZ,* or *IL12A* but not with exon10 of *CXXC5* (Supplementary Information Fig. S10a).

Our analyses for the simultaneous presence of CXXC5 and MeCP2 with the promoters of genes we tested by sequential-ChIP from cells co-synthesizing 3F-CXXC5 and HA-MeCP2 were inconclusive. However, ChIP-qPCRs with the use of equal aliquots of extracts from cells synthesizing 3F-CXXC5 and HA-MeCP2 with the Flag or the HA antibody suggested the presence of CXXC5 and MeCP2 on the promoter region of *HDAC11, NFKBIZ, or IL12A* (data not shown), as we similarly observed in cells transfected with 3F-CXXC5 or HA-MeCP2 alone (Supplementary Information Fig. S10a).

**Figure 8:**
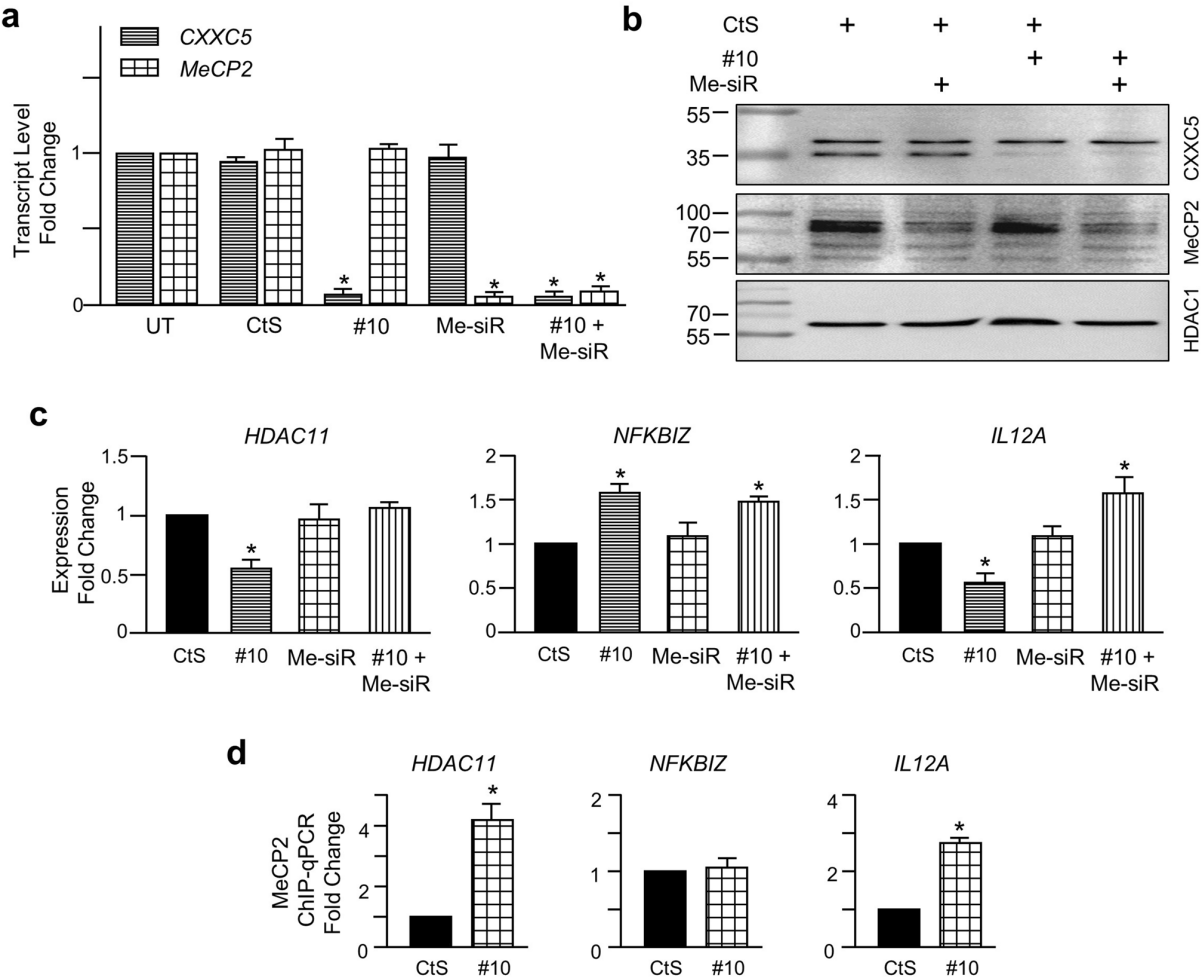
Assessing the interplay between CXXC5 and MeCP2 in CXXC5 target gene expressions. (**a**) MCF7 cells were untransfected (UT), or were transiently transfected with CtS, siRNA specific for CXXC5 (#10), and/or siRNA pool for MeCP2 (Me-SiR) for 48 h. To equalize the total amount of siRNA (20 nM) used in co-transfection experiments, 10 nM gene-specific siRNA was used together with 10 nM CtS. cDNA generated from total RNA were subjected to qPCR. The *RPLP0* expression was used for normalization. Results, the mean ± S.E. of three independent determinations, depict fold change in comparison with transcript levels of CXXC5 or MeCP2 of UT, which were set to 1. The asterisk indicates a significant difference. (**b**) Nuclear extracts of transfected cells were also subjected to WB using the CXXC5 or MeCP2 antibody. HDAC1 was probed with an HDAC1-specific antibody. MMs in kDa are indicated. Uncropped blots are presented in Supplementary Information, Figure S11. (**c**) To assess the effect of reduction in CXXC5 and/or MeCP2 levels on gene expressions, MCF7 cells were transfected with CtS, #10, and/or Me-SiR as indicated for 48 h. cDNAs were then subjected to qPCR using primer sets specific to target genes. *RPLP0* expressions were used for normalization. Results as fold change in gene expressions compared to those observed in CtS transfected cells are the mean ± S.E. of three independent determinations. Asterisks indicate significant differences. (**d**) To assess whether alterations in CXXC5 levels affect the MeCP2 loading on target promoters, MCF7 cells transfected with CtS (10 nM) or #10 (10 nM) for 48 h were subjected to ChIP using IgG or a MeCP2 antibody. Recovered DNAs were subjected to qPCR using primer sets for target gene promoters. Results, normalized to IgG, depict fold changes compared to CtS, which was set to 1.

These results imply that an interplay between CXXC5 and MeCP2 could contribute to the expression of *HDAC11*, *NFKBIZ*, or *IL12A*. To test this possibility, we examined the alterations in the expression of *HDAC11*, *NFKBIZ*, or *IL12A* in MCF7 cells transiently transfected with a control siRNA (CtS), a siRNA specific to *CXXC5* (#10), and/or a siRNA pool specific to MeCP2 (Me-siR). Results revealed that CtS did not affect the transcript or protein levels of CXXC5 or MeCP2. #10 specifically repressed the transcript and protein levels of CXXC5 without affecting those of MeCP2 (Fig. 8a,b; Supplementary Information, Fig. S11). Re-probing the membrane with an antibody specific to MeCP2, which also immunoprecipitates endogenous MeCP2 in MCF7 cells (Supplementary Information, Fig. S12), indicated that Me-siR specifically suppressed the transcript and protein levels of MeCP2. Co-transfection of #10 and Me-siR led to suppression of the transcript and protein levels of both CXXC5 and MeCP2 (Fig. 8a,b; Supplementary Information, Fig. S11).

Repression of CXXC5 and MeCP2 protein levels resulted in the alteration of *HDAC11*, *NFKBIZ*, or *IL12A* expression. Transfection of cells with #10 alone attenuated the expression of *HDAC11* and *IL12* but augmented the *NFKBIZ* expression. Me-siR alone, on the other hand, did not affect the expression of *HDAC11*, *IL12A*, or *NFKBIZ*. But when co-transfected, Me-siR counteracted the repressive effect of #10 on *HDAC11* expression, prevented and further augmented the expression of *IL12A* without affecting the transcript levels of *NFKBIZ* enhanced by #10 (Fig. 8c). To assess whether modulations in gene expressions were due to changes in the extent of MeCP2 interactions with the promoter region of *HDAC11*, *NFKBIZ,* or *IL12A*, we carried out ChIP-qPCR from MCF7 cells transfected with CtS or siRNA#10 for 48 h. Cells were then subjected to ChIP using the MeCP2 antibody or an isotype-matched IgG. Results, depicted as fold changes compared to CtS following normalization to IgG, revealed that the association of MeCP2 with the promoter regions of *HDAC11* and *IL12A* increases but does not change at the *NFKBIZ* promoter (Fig. 8d) or Exon10 of *CXXC5* as the control (Supplementary Information, Fig. S10b).

These results collectively suggest that an interplay between CXXC5 and MeCP2 at promoter regions contributes to the modulation of a subset of CXXC5 target gene expressions. We also observed that the methylation state of the promoter region of *HDAC11*, *NFKBIZ,* or *IL12A* is not affected by the reduction of CXXC5 levels (Supplementary Information Fig. S13b). This implies that modulation of a subset of gene expressions by the integrated effects of CXXC5 and MeCP2 may not require changes in DNA methylation.

#### Correlation analysis between mRNA expressions of CXXC5 and MeCP2 in breast cancer patients

To assess whether there is a correlation between the *CXXC5* and *MeCP2* expressions in clinical settings particularly in breast cancer patients that could highlight the importance of our observations, we used the GEPIA (Gene Expression Profiling Interactive Analysis) webserver for the expression analyses of *CXXC5* and *MeCP2* based on paired normal tissue and tumor samples from the TCGA and healthy tissue samples from the GTEx databases^65^. We found that the mean gene expression profile of *CXXC5* is higher in breast tumor samples compared to mammary tissue together with paired normal breast tissue samples (Supplementary Information Fig. S14a), while the mean expression of the MeCP2 gene does not show significant variations between breast tumor and paired normal tissue samples (Supplementary Information Fig. S14b). We also found that although CXXC5 expression is significantly deregulated, there is no strong correlation between the *CXXC5* and *MeCP2* expressions in healthy mammary tissue in paired normal breast samples and breast tumors (Supplementary Information Fig. S14c–e), neither in many other tissue tumors including acute myeloid leukemia, brain lower grade glioma, glioblastoma multiforme, sarcoma, prostate adenocarcinoma, and testicular germ cell carcinoma (Supplementary Information, Fig. S15).

## Discussion

Our findings indicate that the proximity interaction partners of CXXC5 identified with BioID in MCF7 cells encompass proteins involved in DNA structural changes, DNA modifications, chromatin remodeling, chromatin/histone modifications, and RNA processing as well as transcription factors and transcription co-regulatory proteins. Since CXXC5 lacks an intrinsic enzymatic activity or a transcription regulatory function but is a preferential unmethylated CpG dinucleotide binder through its CXXC domain^4,21,66,67^, our results together with the previous studies^12,22–24,26,27,29–31,33–36^ suggest that CXXC5 could act as a molecular scaffold for the regulation of gene expressions leading to cellular proliferation, differentiation, and death in a cell context-dependent manner^11–13,15,21,22,24,28–30^. This prediction presupposes the binding of CXXC5 to unmethylated DNA. However, it is also likely that CXXC5 without interacting with DNA associates directly or indirectly as a part of a protein complex with various transcription factors and/or DNA/chromatin binders to modulate gene expressions. Previous studies^12,37^, as we find here, also indicate that the DNA binding feature of the CXXC domain is independent of the ability to interact with protein partners, as the DNA binding CXXC mutants retain interactions with Dvl1^37^ or HDAC1^12^. These findings indicate bi-functionality for the CXXC domain of CXXC5: DNA and protein interactions. Moreover, the interaction of CXXC5 with KMT1A (SUV39H1) was reported to occur through a central region (aa 101–200) of CXXC5. This, together with our observation that the efficiency of interactions of the CXXC domain with protein partners could be modulated by a centrally located region (aa 100–150) of CXXC5, suggests that in addition to the CXXC domain, CXXC5 may contain distinct protein interaction regions and/or surfaces emerging from inter/intra-molecular allosteric interactions that could control associations or interaction affinities with protein partners. This, in turn, implies that dynamic conformational fluctuations of CXXC5 with a highly disordered amino-terminus region^21^ as results of DNA binding, protein–protein interactions, and/or post-translational modifications in a cellular environment are critical for diverse functional features of the protein in a signal- and cell type-specific manner.

Nuclear lamina (NL) is an interwoven structure composed of lamins and lamin-associated proteins of the inner nuclear membrane (INM). NL and proteins resident in the INM form a dynamic network that regulates chromatin organization, cell cycle regulation, DNA replication, DNA repair, cell differentiation, and death^68,69^. The NL is composed of Lamin A and C, which are the two major splice variants of a single gene (*LMNA*), as well as Lamin B1 and B2 encoded by *LMNB1* and *LMNB2,* respectively^68,69^. Lamins interact with chromatin either directly or indirectly through chromatin-binding proteins. Lamin B1/B2 interacts with LBR (Lamin B Receptor) and HP1 (heterochromatin protein 1) associated with heterochromatin. On the other hand, Lamin A/C interacts with members of the LEM-Domain family (LEMD) proteins, including LAP2A/B and EMD through a nucleoplasmic adaptor protein BANF1 (barrier-to-autointegration factor, BAF), which binds to histones and DNA associated with both heterochromatin and euchromatin. We observed here that the proximity interaction partners of CXXC5 include EMD and LAP2A. We found that CXXC5 interacts with EMD but not with LAP2A, which resides in the nucleoplasm rather than INM due to the lack of a membrane-spanning domain present in INM proteins. Moreover, we observed that CXXC5 retains interactions with EMD lacking the LEM domain required for BANF1 binding consequently chromatin interactions (Supplementary Information, Fig. S16). It is therefore plausible that CXXC5 as an unmethylated CpG binder could anchor DNA to the INM through interactions with EMD, thereby providing a local chromatin environment critical for the modulation of target gene expressions in a nuclear activity-dependent manner.

Similarly, the interaction of CXXC5 with MAZ, a transcription factor with six Cys2His2-type zinc finger motifs at the carboxyl-terminus that binds to the permutation of the canonical GGGAGGG DNA sequence, could be critical for reciprocal recruitment, or assisted loading, to regulatory sites of target genes. This could result in coordinated recruitment of coregulatory proteins, including epigenetic factors, exemplified with nucleosome-remodeling factor (NURF) subunit BPTF as the proximity interaction partner of CXXC5 (Supplementary Information, Table S1) and the interactor of MAZ^70^ for the regulation of target gene expressions.

DNA methylation is one of the mechanisms of gene silencing and it mostly occurs in CpG dinucleotides of the genome. The effect of DNA methylation on gene expression could be refractory and/or permissive, depending on the genomic region. DNA methylation at promoters represses transcription of genes, while DNA methylation of intra/intergenic regions with different degrees of CpG density appears to correlate with gene expression^71^. DNA methylation is intimately associated with histone modifications, leading to integrated epigenetic processes for the alteration of chromatin architecture and the subsequent modulation of gene expressions. Methylated DNA is specifically recognized by MBPs, which belong to three distinct structural families: the Methyl-binding domain (MBD), the Methyl-CpG binding zinc fingers, and the SRA (SET- and RING-associated) domain proteins^72^. As a member of the MBD family, MeCP2 functions as a genome-wide transcriptional modulator. Mutations in the X-linked MeCP2 gene lead to a severe neurodevelopmental disorder, Rett syndrome^73^. MeCP2 readily binds both methylated and unmethylated DNA. MeCP2 through the MBD domain interacts with methylated/hemimethylated CG dinucleotides as well as methylated/hemimethylated CAC tri-nucleotides. MeCP2 also interacts with methylated cytosines in the non-CG context (mCH, where H = A, C, or T) as well as nucleosomes^74–82^. The MBD domain of MeCP2 also binds to unmethylated 5'-CAC/GTG-3' motif-containing DNA with an affinity comparable to methylated DNA^74^. In addition, the ID, TRD, and CTD domains of MeCP2 exhibit methylation-independent DNA binding capabilities^83^, and AT-hook-like domains within the ID, TRD, and CTD alpha domains of MeCP2 bind to the minor groove of AT-rich DNA^84^.

These methylation-dependent and independent DNA binding capabilities, together with the ability of MeCP2 to bind to DNA cooperatively and to induce DNA bridging and looping^83,85,86^, are suggested to allow MeCP2 to interact with different sites on DNA simultaneously, thereby contributing to genome-wide chromatin organization^52,87^. Upon binding to DNA/histones at gene regulatory regions, MeCP2 could directly hinder the binding of transcription factors to cognate response elements or indirectly through sequential and ordered recruitment of distinct members of the chromatin remodeling complexes to generate a chromatin state refractory for transcription. Moreover, MeCP2 suppresses transcription by binding to methylated cytosine within transcribed regions of gene bodies thereby impeding transcriptional elongation^88^. Besides transcription attenuation/repression, MeCP2 also functions as an activator/enhancer of gene expressions by engaging transcriptionally active promoters^81^ and recruiting coactivator proteins, including CREB1 (CAMP responsive element binding protein 1)^82^ and MYCN (MYCN Proto-Oncogene, BHLH Transcription Factor)^89^.

We found here that CXXC5 interacts with MeCP2, and the interaction involves the carboxyl-terminal CXXC domain of CXXC5 independently of its ability to bind to DNA and the carboxyl-terminus of the WW domain (CTD β domain) of MeCP2. We observed that CXXC5 and MeCP2 are associated with the promoter region, located within a CGI, of *HDAC11*, *IL12A*, or *NFKBIZ* gene. Furthermore, we observed that an interplay between CXXC5 and MeCP2 at *HDAC11*, *IL12A*, and *NFKBIZ* promoter regions is critical for the magnitude of gene expressions independently of DNA methylation. These observations imply that the integrated effects of CXXC5 and MeCP2 are important for the transcriptional output of some of the CXXC5 target genes. However, underlying mechanistic features of these integrated effects remain, at this juncture, speculative. The interaction of the DNA-bound CXXC5 with MeCP2 may lead to the recruitment of chromatin remodelers and/or co-regulatory proteins to fine-tune, augment or repress, the expression of a subset of target genes. One anticipated result would then be a decrease in the association of CXXC5 with DNA when CXXC5 is knockdown should reduce the extent of DNA association of MeCP2. In contrast, we observed an increased presence of MeCP2 at the promoter regions of *HDAC11* and *IL12A* but not at the *NFKBIZ* gene promoter. This suggests that the binding of CXXC5 to DNA restricts the DNA association of MeCP2 at *HDAC11* and *IL12A* promoters independent of CXXC5 interactions, thereby increasing the repressive potential of MeCP2 for transcription when the CXXC5 levels are reduced. It is, therefore, possible that the DNA-bound CXXC5 independently of MeCP2 is involved in the magnitude of the gene expression through interactions with various chromatin modelers and/or transcription co-regulators or preventing the binding of various transcription factors to cognate response elements at the target gene promoters to establish a transcription state. It is also possible that the recruitment of MeCP2 by the DNA-associated CXXC5 or the interaction of CXXC5 with the DNA/histone-bound MeCP2 constrains the modulatory effects of MeCP2 for gene expressions by sterically blocking the ability of either interaction partner to recruit co-regulatory complexes. This could, when the levels of both proteins are reduced, result in no change, as observed with *HDAC11*, or in augmentation by causing accessibility to other TFs, as in the case of *IL12A*, in the level of gene expression. We also observed that the knockdown of CXXC5 leads to an enhanced *NFKBIZ* expression without affecting the extent of the association of MeCP2 with the *NFKBIZ* promoter. The repression of MeCP2 levels, on the other hand, did not affect the *NFKBIZ* expression. This suggests that the DNA-associated CXXC5 recruits, or assists the loading of, MeCP2 to repress the *NFKBIZ* expression. But we also observed that the expression of *NFKBIZ* remains elevated when both CXXC5 and MeCP2 protein levels are suppressed. This raises the possibility that the binding of various TFs to exposed cognate binding sites as a result of reduced levels of CXXC5, and thereby the associated MeCP2, increases the *NFKBIZ* expression. Deciphering possibilities involved in target gene expressions could further shed light on the function of CXXC5 in cellular events.

In summary, the proximity interaction analysis we employed here indicates that although lacks an intrinsic transcription regulatory activity or an enzymatic function, CXXC5 as an unmethylated CpG binder interacts with various DNA/chromatin modelers and transcription factors/co-regulators and is involved in the modulation of target gene expressions. While constituting a prelude for the identification of interaction partners of CXXC5, our findings here provide a basis for a better understanding of the regulatory mechanisms of CXXC5-mediated cellular processes in response to intrinsic and extrinsic stimuli in a lineage-specific manner in both physiology and pathophysiology that could offer clinical benefits.

## Materials and methods

### Cell culture and transfection

MCF7 and HEK293 cells were cultured in phenol red-free, high glucose (4.5 g/L) containing Dulbecco’s modified Eagle’s medium (DMEM, Lonza, Belgium, BE12-917F) supplemented with 10% fetal bovine serum (FBS, Lonza), 1% L-Glutamine (Lonza, BE17-605E) and 1% Penicillin/Streptomycin (Lonza, Belgium) as described previously^19,21,90^. MCF7 or HEK293 cells were transiently transfected with Turbofect transfection reagent (R0533; ThermoFisher, Waltham, MA, USA) for 48 h if not otherwise specified. Protein concentrations in extracts were assessed with a Bradford protein assay kit (Bio-Rad Life Sciences; 5000001). Restriction and DNA modifying enzymes were obtained from New England Bio-Labs (Beverly, MA, USA) or ThermoFisher. Chemicals were obtained from Sigma-Aldrich (Germany) or ThermoFisher. Pageruler Prestained Protein Ladder (ThermoFisher; 26616) or Pageruler *Plus* Prestained Protein Ladder (ThermoFisher; 26620) was used as the molecular mass (MM) marker. In all PCR-based approaches, at least two distinct primer sets, and their combinations, designed with the PrimerQuest Tool of Integrated DNA Technologies (IDT; https://www.idtdna.com/pages/tools/primerquest) were initially used for testing their efficiencies in amplifying template DNA (genomic, cDNA, or plasmid) under various conditions including varying temperatures without or with various DMSO concentrations. Based on PCR results, a primer set giving the best amplification efficiency was used in experiments and reported in Supplementary Information, Table S2.

### Generation and functional analyses of protein components of BioID

To generate protein components of BioID, the 3xFlag-CXXC5 (3F-CXXC5) cDNA obtained with PCR using the wild-type CXXC5 cDNA^19^ as the template was genetically fused to the 5’ end of a sequence encoding the BirA*-HA cDNA present in the pcDNA expression vector, pcDNA3.1-BirA*(R118G)-HA obtained from Addgene (36047). To generate BirA*(R118G)-HA cDNA with the translation initiation codon encoding methionine within the context of Kozak sequence (CGCCATG), we used PCR with primers and BirA*(R118G)-HA cDNA as the template. The cDNA was then cloned into the pcDNA3.1 vector and sequenced to ensure the fidelity of the encoding sequences. To assess the synthesis and intracellular location of the protein components of BioID, we carried out western blot (WB) and immunocytochemistry (ICC) analyses in transiently transfected MCF7 cells derived from a breast adenocarcinoma. The expression vector pcDNA3.1 bearing none, the BirA*-HA, the 3F-CXXC5, or the 3F-CXXC5-BirA*-HA cDNA were transiently transfected into MCF7 cells for 24 h. Cells were then treated without or with 50 μM biotin (Sigma-Aldrich; B4639) and 1 mM ATP (Adenosine 5’-triphosphate disodium salt hydrate, Sigma-Aldrich; A2283) for 16 h followed by ICC and WB.

For WB analyses, cellular extracts (100 µg) using M-PER Mammalian Protein Extraction Reagent (ThermoFisher; 78,501) were subjected to SDS-%10PAGE followed by transfer onto a PVDF membrane (Advansta, WesternBright PVDF-CL, L-08008–001) using a wet transfer system at 100 V for 1 h. The membrane was blocked with buffer containing 5% skim milk (Bio-Rad, 170-6404) in 0.1% Tris Buffered Saline-Tween (TBS-T) for overnight at 4 °C followed by the incubation with the Flag antibody (SigmaAldrich; F1834; 1:1000 dilution), the HA (Abcam, ab9110; 1:1000 dilution) or the Biotin (Abcam ab53494; 1:200 dilution) antibody in the blocking buffer for 1 h at room temperature. The membrane was then washed extensively with 0.1% TBS-T followed by incubation with the blocking buffer containing an HRP conjugated goat anti-mouse IgG secondary antibody (Advansta, R-05071-500; 1:4000 dilution) for the Flag antibody or an HRP conjugated goat anti-rabbit IgG secondary antibody (Advansta, R-05072-500; 1:4000 dilution) for the HA or the Biotin antibody for one hour at room temperature. After an extensive wash with 0.1% TBS-T, the membrane was incubated for two minutes with WesternBright ECL reagent (Advansta, K-12045-D50) at 1:1 luminol-enhancer reagent:peroxide ratio. Visualizations were carried out using the ChemiDoc MP system (Bio-Rad, 1708280), and images were analyzed with Image Lab software (Bio-Rad).

For ICC, MCF7 cells (5 × 10^4^) grown on coverslips in 12-well tissue culture plates were transiently transfected for 48 h. Cells were then washed with PBS and fixed with 3.7% formaldehyde in PBS for 30 min. The cells were permeabilized with 0.4% Triton-X-100 in PBS for 10 min followed with the incubation containing 10% normal goat serum in PBS for the HA or the Biotin antibody or 10% BSA in PBS for the Flag antibody to block the non-specific antibody binding for 1 h. Cells were then incubated with the HA (Abcam, ab9119; 1:500 dilution), the Flag (Sigma Aldrich, F-1804; 1:250 dilution), or Biotin (Abcam ab53494; 1:100 dilution) antibody in the corresponding blocking buffer for 2 h. Cells were subsequently washed with PBS and incubated with a goat anti-mouse IgG H&L (Alexa Fluor 488; green-fluorescent dye; ab150113; 1:1000 dilution) for the Flag antibody, a goat anti-rabbit IgG H&L (Alexa Fluor 594; red-fluorescent dye; ab150077; 1: 1000 dilution) for the HA antibody or the Biotin antibody at room temperature for 1 h. The cells were rinsed in PBS and mounted onto glass slides with a mounting medium containing DAPI (4,6-diamidino-2-phenylindole; blue-fluorescent stain) for nuclear staining (Abcam, ab104139). Images were viewed and captured with the Nikon Eclipse 50i Fluorescence Microscope.

### Proximity-dependent biotinylation (BioID) assay

To identify the proximity interaction partners of CXXC5, we carried out the BioID assay. MCF7 cells (2.5 × 10^6^/10 cm^2^ culture dish of total 10 culture dishes) grown for 48 h were transiently transfected with the expression vector pCDNA3.1 bearing none, the BirA*-HA, or the 3F-CXXC5-BirA*-HA cDNA for 24 h. Cells were then treated with 50 μM Biotin 1 mM ATP for 16 h. Cells were collected and washed with cold PBS and then lysed at room temperature in lysis buffer [50 mM Tris, pH 7.4; 500 mM NaCl; 0.4% SDS; 5 mM EDTA; 2% TritonX; 1 mM DTT with freshly added protease (Roche; 5892970001) and phosphatase (Roche; 4906845001)] inhibitors. Cell lysates were sonicated for a total of 7.5 min (with 5-s pulse and 10-s rest in between pulses) and centrifuged at 7500 rpm for 10 min at 4 °C. The supernatant was incubated with 50 µl Streptavidin magnetic beads (NEB, S1420S) overnight. Beads were collected and washed twice with Wash Buffer I (2% SDS in dH_2_O) for 10 min. Beads were washed once with Wash Buffer II (2% deoxycholate; 1% TritonX; 50 mM NaCl; 50 mM HEPES pH 7.5; 1 mM EDTA) for 10 min, once with Wash Buffer III (0.5% NP-40; 0.5% deoxycholate; 1% TritonX; 500 mM NaCl; 1 mM EDTA; 10 mM Tris pH 8.0) for 10 min, and once with Wash Buffer IV (50 mM Tris, pH 7.4; 50 mM NaCl) for 30 min, respectively, and all washing steps were carried out at room temperature. 10% of bound proteins were eluted from the streptavidin beads with 50 µl of Laemmli-DTT sample buffer containing 500 nM D-Biotin for WB analyses using the biotin antibody (Ab533494) and the remaining samples were subjected to Mass Spectrometry (MS) analyses.

### Protein identification by mass spectrometry

MS analyses were carried out at the Koç University Proteomic Facility (Istanbul, Turkey). The protein-bound streptavidin beads were washed with 50 mM NH_4_HCO_3_, followed by reduction with 100 mM DTT in 50 mM NH_4_HCO_3_ at 56 °C for 45 min, and alkylation with 100 mM iodoacetamide at RT in the dark for 30 min. MS Grade Trypsin Protease (Pierce) was added onto the beads for overnight digestion at 37 °C (enzyme: protein ratio of 1:100). The resulting peptides were purified using C18 StageTips (ThermoFisher). Peptides were analyzed by online C18 nanoflow reversed-phase HPLC (2D nanoLC; Eksigent) linked to a Q-Exactive Orbitrap mass spectrometer (ThermoFisher). The data sets were searched against the human SWISS-PROT database version 2014_08. Proteome Discoverer (version 1.4; ThermoFisher) was used to identify proteins. The final protein lists were analyzed using the STRING v10.5^91^ and DAVID^92^ databases.

### Validation of interaction partners of CXXC5

#### Cloning

Based on BioID results, we cloned the cDNA for MeCP2 (Methyl CpG Binding Protein 2), MAZ (MYC Associated Zinc Finger Protein), or EMD (Emerin) into the pcDNA 3.1(-) vector bearing in-frame sequences at the 5’-end of the multiple cloning site that encode for an amino-terminally located 3xFlag or HA tag. The cDNA for MeCP2 was kindly provided by Dr. K. Miyake, University of Yamanashi, Japan. The EMD (HsCD00324605) or the MAZ (HsCD00377948) cDNA was obtained from the Harvard Plasmid (https://plasmid.med.harvard.edu). The MAZ cDNA encodes an amino-terminally truncated variant that uses a methionine residue at position 230 of the full-length MAZ (477 amino acid) as the first methionine, which results in the synthesis of a 248 amino acid long truncated MAZ protein with an estimated MM of 28 kDa (MAZ_ΔN_). Using cDNAs as templates we generated PCR amplicons by PCR and inserted them into the pcDNA 3.1(-) expression vector. We also cloned a MAZ cDNA encoding the full-length protein with an estimated MM of 51.1 kDa using a cDNA library from MCF7 cells and PCR into the 3xFlag- or HA-tagged pcDNA 3.1(-) vector. Similarly, EMD lacking the LEM domain, EMD_ΔLEM_, residues of 1 through 47^45^ was cloned the 3xFlag- or HA-tagged pcDNA 3.1(-) vector.

To identify a sub-region(s) of CXXC5, we generated cDNAs encoding amino and/or carboxyl-terminally truncated CXXC5 proteins. To ensure that some CXXC5 variant proteins lacking the nuclear localization signal located at the carboxyl-terminus CXXC domain localize to the nucleus as the full-length CXXC5 we inserted sequences generated by PCR using the CXXC5 cDNA as the template to encode an NLS derived from the SV40 T antigen^54^ between sequences encoding the Flag epitope and a CXXC5 variant.

All constructs were sequenced for the fidelity of encoding sequences. Tag and Primer sequences are given in Supplementary Information, Table S2.

#### ICC

HEK293 cells (2.5 × 10^4^) plated on coverslips in a well of 12-well tissue culture plates were grown for 48 h. Cells were then transiently transfected with expression vectors bearing the 3F-CXXC5 (or HA-CXXC5) cDNA alone or together with the HA or 3F tagged EMD, MAZ, or MeCP2 cDNA using Turbofect transfection reagent (Thermo Scientific, R0532). 48 h after transfections, cells were processed for ICC as described above using the Flag and/or HA antibodies followed by Alexa Fluor conjugated secondary antibodies for visualization with a Nikon Eclipse 50i Fluorescence Microscope. ImageJ software was used for image analysis.

#### WB

HEK293 cells (15 × 10^4^) plated on six-well tissue culture plates for 48 h were transiently transfected with the expression vector bearing the 3F-CXXC5 (or HA-CXXC5) cDNA alone or together with the HA- or 3F-tagged EMD, MAZ, or MeCP2 cDNA using Turbofect transfection reagent (Thermo Scientific, R0532). Cells were then processed for WB as described above.

#### Co-Immunoprecipitation (Co-IP)

Transiently transfected HEK293 cells in six-well plates were collected with trypsin and lysed with NE-PER (ThermoFisher; 78,833) that contained freshly added protease and phosphatase inhibitors. The protein concentration of lysates was measured by the Bradford Protein Assay. To block non-specific protein binding to magnetic beads, 500 μg lysates were incubated with non-specific IgG (5 μg) together with 25 μl Protein A and G conjugated magnetic beads at 4 °C for 1 h. Lysates in 1.5 ml centrifuge tubes were then applied to a magnetic field for 30 s to pull the beads to the side of the tube. The supernatant was transferred to a clean 1.5 ml microcentrifuge tube and beads were discarded. The pre-cleared lysates were subsequently incubated with a 5 µg HA or Flag antibody at 4 °C overnight and followed by the addition of 25 μl Protein A and G conjugated magnetic beads at 4 °C for 1.5 h Beads were then washed two times with 500 μl IP buffer composed of 150 mM NaCl, 10 mM HEPES pH 7.5, 10 mM MgCl_2_, 0.5% Igepal, protease inhibitor, and phosphatase inhibitors. Bead pellets were resuspended in 30 μl of 2xLaemmli-SDS buffer [187.5 mM Tris–HCl (pH 6.8), 6% (w/v) SDS, 30% glycerol, 150 mM DTT, 0.03% (w/v) bromophenol blue, 2% β-mercaptoethanol] and incubated at 95 °C for 5 min. Samples were then applied to a magnetic field for 30 s and supernatants were subjected to SDS-10%PAGE for WB analysis using the Flag or the HA antibody followed by the HRP conjugated VeriBlot for IP Detection Reagent (Abcam, ab131366).

#### Proximity ligation assay (PLA)

Duolink In Situ Red Starter kit (Sigma-Aldrich) was used according to the manufacturer’s instructions. In brief, HEK293 cells (2.5 × 10^4^) were grown on glass coverslips in a well of a 12-well tissue culture plate for 48 h. Cells were then transfected with the expression vectors bearing the 3F-CXXC5 and HA-MeCP2 cDNA. After transfection, cells were fixed with 3.2% PFA in PBS for 10 min, permeabilized with 0.1% Triton-X for 5 min, and then blocked with Duolink blocking solution at 37 °C for 30 min. Cells were subsequently probed with the Flag (1:500) and/or the HA (1:250) antibody overnight at 4 °C. Cells were then treated with fluorescent probes for 1 h at 37 °C. Cells were washed in wash buffer A for 10 min at RT and incubated with secondary antibodies conjugated to plus and minus PLA probes for 1 h at 37 °C. After repeating the washing step with wash buffer A for 10 min at RT, cells were incubated with the ligase for 30 min at 37 °C. After another washing cycle with wash buffer A, cells were incubated with the polymerase in the amplification buffer for 100 min at 37 °C. Finally, the cells were washed in 1X wash buffer B for 20 min and then with 0.01X wash buffer B for 1 min at RT. Duolink In Situ Mounting media with DAPI was used for nuclear staining. Images were captured with a Nikon Eclipse 50i Fluorescence Microscope. ImageJ software was used for image analysis.

#### Chromatin Immunoprecipitation (ChIP), ChIP-WB and ChIP-qPCR

ChIP was carried out as described^19,93,94^. In all ChIP experiments (ChIP-WB or ChIP-qPCR), transfected cells (10 × 10^6^) were fixed with 1% paraformaldehyde at room temperature (RT) for 10 min with gently shaking. Glycine was then added to a final concentration of 125 mM to quench the paraformaldehyde at RT for 5 min with gently shaking. Cells were washed with PBS twice and collected in 5 ml PBS with a cell scraper. The pelleted cells were lysed with ChIP lysis buffer (1% SDS, 10 mM EDTA, 50 mM Tris–HCl, pH 8.1) and sonicated to shear DNA to lengths between 200 and 1000 bp. Cell debris was then pelleted and the supernatant was collected. The supernatant was diluted with ChIP dilution buffer (0.01% SDS, 1.1% Triton X-100,1.2 mM EDTA, 16.7 mM Tris–HCl, pH 8.1, 167 mM NaCl), supplemented with protease inhibitor (Roche). To preclear, diluted samples were incubated with 30 µl Protein A (NEB #S1425S) and 30 µl Protein G (NEB #S1430S) magnetic beads with rotation for 1 h at 4 °C. The beads were then pelleted with a magnetic separator and the supernatant was collected. 10% of supernatant was set aside as the input control. The Flag, the HA antibody, or a species-specific IgG (10 µg) were then added into the supernatant and incubated for 1 h at 4 °C with rotation. Protein A/G magnetic beads (60 µl) were then put into the mixture overnight at 4 °C with rotation. The mixture was pelleted with a magnetic separator. Immunoprecipitates were sequentially washed once each with wash buffers containing low salt (0.1% SDS, 1%Triton X-100, 2 mM EDTA, 20 mM Tris–HCl, pH 8.1, 150 mM NaCl) high salt (0.1% SDS, 1%Triton X-100, 2 mM EDTA, 20 mM Tris–HCl, pH 8.1, 500 mM NaCl), and lithium chloride (0.25 M LiCl,1% NP40, 1% deoxycholate, 1 mM EDTA, 10 mM Tris–HCl, pH 8.1) followed by two washes with Tris–EDTA buffer (10 mM Tris–HCl, 1 mM EDTA,pH 8.0).

For ChIP-WB, immunoprecipitates following washes were directly dissolved in 40 µl 6XLaemmli buffer (375 mM Tris–HCl pH 6.8, 6% SDS, 4.8% Glycerol, 9% 2-Mercaptoethanol, 0.03% Bromophenol blue) and were boiled for 10 min. Beads were removed with a magnetic stand and the supernatants were subjected to SDS-8%PAGE followed by WB.

For ChIP-qPCR, immunoprecipitates after washes were resuspended with a ChIP elution buffer (10 mM Tris–HCl pH 8.0, 1 mM EDTA) followed by the addition of NaCl (to the final concentration of 300 mM) to de-crosslink protein-DNA interactions. Samples were then incubated at 37 °C for 1 h for RNase treatment and followed by incubation at 65 °C for 4 h with Proteinase K treatment (10 mg/ml). DNA was then recovered with phenol:chloroform:isoamyl alcohol (25:24:1) followed by ethanol precipitation of DNA for qPCR.We also carried out ChIP for endogenous MeCP2 using a MeCP2-specific antibody (Proteintech Group, Inc., Rosemont, IL, USA; 10861-1-AP) in MCF7 cells transiently transfected with 10 nM of a scrambled siRNA (AllStar, CtS) for 48 h. For ChIP-WB, samples from ChIP carried out as described above were resuspended in 40 µl of 2xLaemmli-SDS buffer and incubated at 95 °C for 10 min. Supernatants were subjected to SDS-8%PAGE for WB analysis using the HA antibody. For ChIP-qPCR, following de-crosslinking and protein digestion, DNA was recovered with phenol:chloroform:isoamyl alcohol (25:24:1) followed by ethanol precipitation and was subjected to qPCR using primers specific for the promoter region of HDAC11, NFKBIZ, IL12A, or the Exon10 of *CXXC5* as a negative control (Supplementary Information, Table S2).

qPCR results were normalized using percent (%) of input approach^90^ and depicted as fold changes compared to CtS following normalization to IgG.

#### siRNA transfections and RT-qPCRs

For siRNA transfection, MCF7 cells in 6-well tissue culture plates were transiently transfected with the HiPerfect transfection reagent (Qiagen) using 10 nM a scrambled siRNA (AllStar, CtS), a siRNA specific for *CXXC5* (siRNA#10; FlexiTube GeneSolution, Qiagen), as we described previously^19,21^, and/or a siRNA pool specific to *MeCP2* (sc-35892, SCBT). To equalize the total amount of siRNA (20 nM) used in co-transfection experiments, 10 nM gene-specific siRNA was used together with 10 nM CtS. Isolated total RNA was used for the cDNA synthesis (The RevertAid First Strand cDNA Synthesis Kit, ThermoFisher). The SYBR Green Mastermix (BioRad, Hercules, CA, USA) and gene-specific primers (Supplementary Information, Table S2) were used for qPCR reactions on BioRad Connect Real-Time PCR.

For the normalization of results, we used the expression of *RPLP0* (60S acidic ribosomal protein P0), as we described previously^95^. The relative quantification of gene expressions was assessed with the comparative 2^-ΔΔCT^ method^96^. For qPCR experiments, MIQE Guidelines were followed^97^.

#### Bisulfite PCR

To assess the DNA methylation state, we used bisulfite DNA sequencing. CtS siRNA or siRNA#10 transfected MCF7 cells for 48 h were subjected to genomic DNA isolation by using QIAamp DNA Mini Kit (Qiagen, 51304) according to the manufacturer’s protocol. Bisulfite conversion of 500 ng of isolated gDNA was performed with EZ-DNA Methylation Lightning Kit (Zymo Research, D5030). The bisulfite converted DNA was used as the template for PCR reaction (LongAmp Taq Polymerase, NEB, M0323) using bisulfite converted DNA specific primers designed with MethylViewer^98^. Amplicons were cloned into the pGEM-T vector (Promega, A3600) for sequencing. Sequences were analyzed with the QUMA^99^ tool (http://quma.cdb.riken.jp/).

#### ChIP-seq data analysis

To investigate DNA regions that CXXC5 could interact with, data from a previously carried out CXXC5 ChIP-seq experiment were analyzed^22^. The analyses were carried out with publicly available bioinformatics tools available on the Cancer Genomics Cloud (CGC) (Seven Bridges Genomics, Boston, USA). Briefly, the raw sequencing reads of ChIP-seq experiments (GEO accession: GSE132025) were retrieved from the NCBI Gene Expression Omnibus (GEO) database as sequence read archive (SRA) files. The SRA files were first converted to FASTQ format using the SRA Toolkit fastq-dump tool. The sequenced reads in FASTQ format were aligned on the mouse reference genome version 10 (mm10) using the Burrows-Wheeler Aligner (BWA) bwa-backtrack algorithm specialized for short reads^96^ and the peaks were called using the MACS2 tool version 2.1.1^100^. Both tools are available on the CGC platform as a workflow. We used the default parameters for both tools and used the broad peak calling functionality of MACS2 to identify binding regions.

#### Correlation analysis between mRNA expressions of CXXC5 and MeCP2 in breast cancer patients

To assess the possible correlation between the *CXXC5* and *MeCP2* expressions, we used the GEPIA (Gene Expression Profiling Interactive Analysis) webserver^65^ for the expression analysis of the *CXXC5* and *MeCP2* genes based on paired normal tissue and tumor tissue samples from the TCGA (https://www.cancer.gov/tcga) and healthy breast tissue samples from the GTEx^101^ databases. The gene expression profiles of *CXXC5* and *MeCP2* across all tumor samples and paired normal tissues as well as the correlation between mRNA expressions of *CXXC5* and *MeCP2* in normal and breast tumor samples were analyzed.

### Statistical analysis

Experiments were repeated at least two independent times. Results, where and when appropriate, were presented as the mean ± standard error (S.E.) of three biological replicates. Statistical analyses were performed using a two-tailed unpaired t-test with a confidence interval, minimum, of 95%.

## Supplementary Information

Supplementary Information.
