# Supplementary material for: A prelude to the proximity interaction mapping of CXXC5
Source: Sci Rep. 2021 Sep 2;11:17587. doi: 10.1038/s41598-021-97060-6 (PMC8413330; doi:10.1038/s41598-021-97060-6)
Supplement: Supplementary file 1 — Supplementary Information. [file 41598_2021_97060_MOESM1_ESM.pdf]

Supplementary Information

A prelude to the proximity interaction mapping of CXXC5

Gamze Ayaz[1,4,6*], Gizem Turan[1*], Çağla Ece Olgun[1*], Gizem Kars[1], Burcu Karakaya[1], Kerim Yavuz[1], Öykü Deniz Demiralay[1], Tolga Can[3], Mesut Muyan[1,2,6], and Pelin Yaşar[1,5*]

[1]Department of Biological Sciences, Middle East Technical University, Ankara 06800, Turkey; [2]Cansyl Laboratories, Middle East Technical University, Ankara 06800, Turkey; [3]Department of Computer Engineering Middle East Technical University, Ankara 06800, Turkey

[4]Current Address: Cancer and Stem Cell Epigenetics Section, Laboratory of Cancer Biology and Genetics, Center for Cancer Research, National Cancer Institute, National Institutes of Health, Bethesda, MD, 20892, USA

[5]Current Address: Epigenetics and Stem Cell Biology Laboratory, Single Cell Dynamics Group, National Institute of Environmental Health Sciences, Research Triangle Park, NC 27709, USA

[6]Corresponding Authors: gamze.ayazsen@nih.gov and mmuyan@metu.edu.tr

[*]Equal contributions; should be considered as first authors

Short title: The proximity interaction partners of CXXC5

Keywords: CXXC5, BioID, Proximity interactions, EMD, MAZ, MeCP2

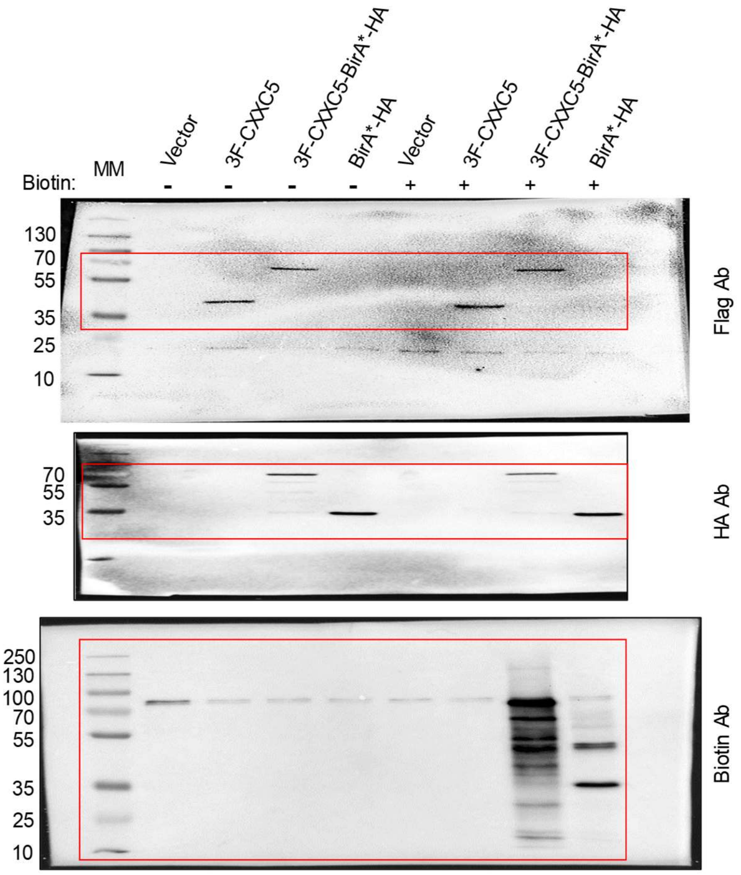

Figure S1. Biotinylation of endogenous proteins in MCF7 cells. MCF7 cells were transiently transfected for 24h with an expression vector (pcDNA3.1) bearing the cDNA for 3F-CXXC5, 3F-CXXC5-BirA*-HA, or BirA*-HA. Cells were then treated without (-) or with (+) biotin (50 µM) and ATP (1 mM) for 16h. Total protein extracts of MCF7 cells were subjected to SDS-10%PAGE followed by WB using the Flag, the HA, or the Biotin antibody followed by an HRP-conjugated goat-anti mouse secondary antibody for the Flag (Advansta R-05071-500) or goat-anti-rabbit secondary antibody for the HA or the Biotin antibody (Advansta R-05072-500). Molecular masses (MM) in kDa are indicated. Cropped images for Fig. 1 are indicated with red boxes.

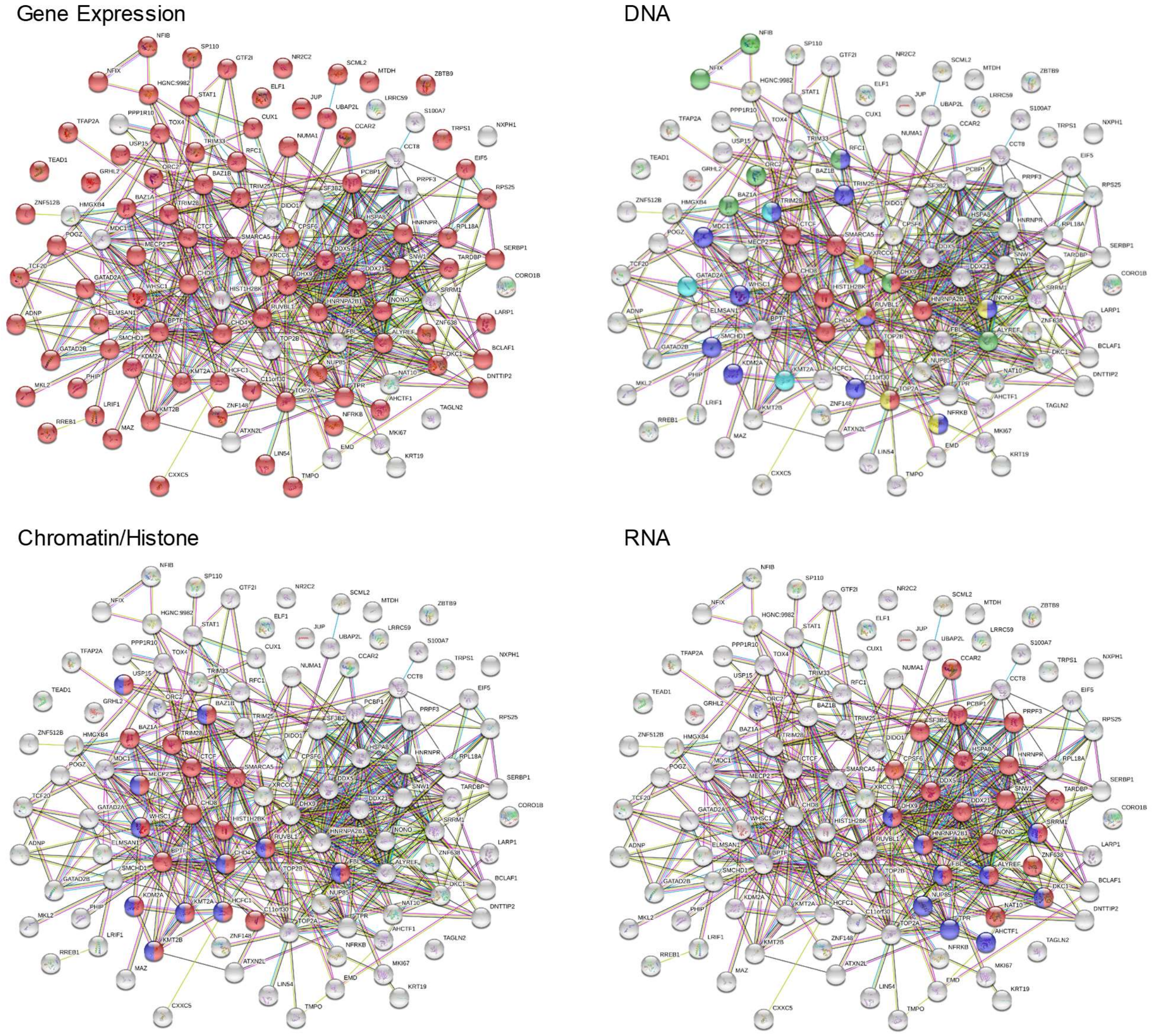

Figure S2. The proximity interaction mapping of CXXC5 with GO term enrichment analyses by the use of the STRING interaction database. Images generated by STRING are shown. The majority of proteins with the proximity interactions are involved in the Regulation of Gene Expressions [(GO:0010468, (red)]. GO terms can further be functionally assigned to DNA [GO:0071103, DNA conformation change (red); GO:0006281, DNA repair (dark blue); GO:0006260, DNA replication (green); GO:0006310, DNA recombination (yellow); GO:0006306, DNA methylation (light blue)]; Chromatin and Histone [(GO:0006325, Chromatin Organization (red); GO:0016570, Histone modifications (dark blue); and, RNA [GO:0006396, RNA processing (red); GO:0031123, GO:0006403, RNA localization (dark blue)] processes.

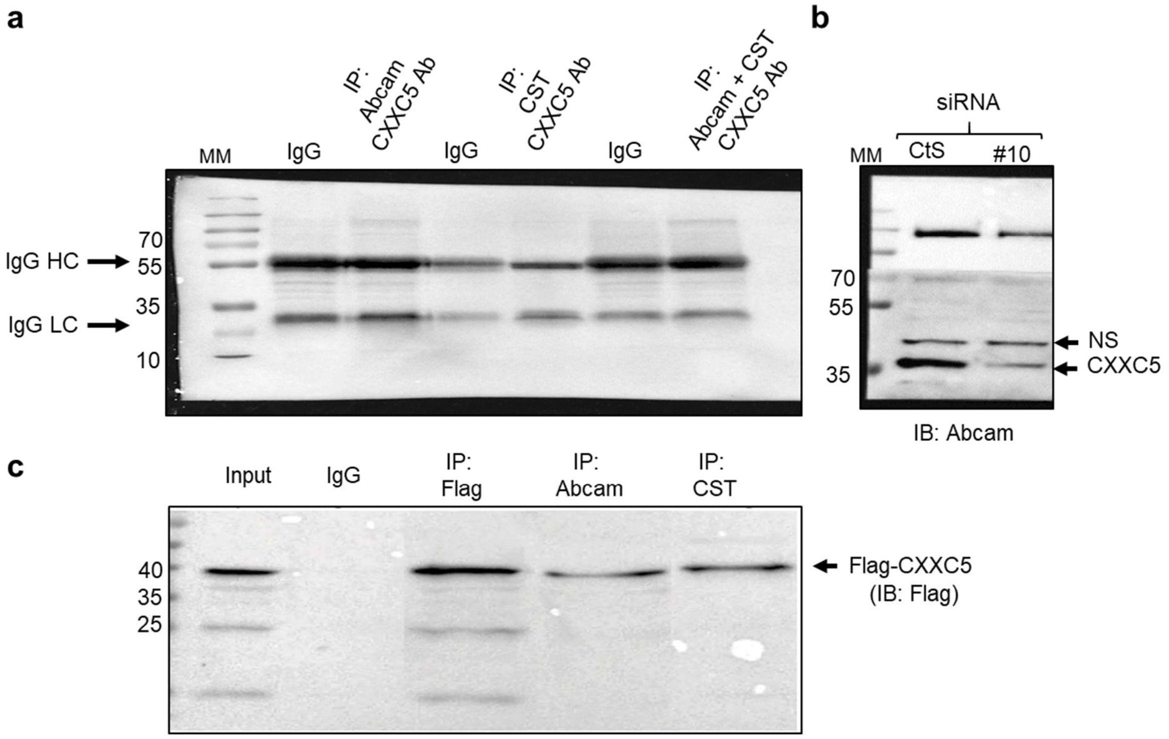

Figure S3. Assessing antibody efficiencies in immunoprecipitation. (**a**) The Abcam antibody (106533) is generated against a 15 aa-long peptide from near the amino terminus of the human CXXC5 protein (https://www.abcam.com/cxxc5-antibody-ab106533.html); whereas, the CST D1O4P antibody is generated against a peptide corresponding to residues surrounding Leu172 of human CXXC5 protein (https://www.cellsignal.com/products/primary-antibodies/cxxc5-d1o4p-rabbit-mab/84546). Nuclear extracts of MCF7 cells (500 µg) were subjected to immunoprecipitation by using 5 µg normal rabbit IgG (IgG), Abcam, or CST (Cell Signaling Technology, D1O4P) antibody. Since the different regions on CXXC5 are used to generate CXXC5 antibodies, the efficiency of immunoprecipitation could be increased by subjecting the nuclear extracts of MCF7 cells (500 µg) to the combination of 2.5 µg Abcam and 2.5 µg CST antibodies. Immunoprecipitants were subjected to SDS-10% PAGE followed by immunoblotting with the Abcam antibody for visualization. (**b**) While both antibodies effectively detect CXXC5 in WB analysis of nuclear extracts of MCF7 cell transiently transfected with the control siRNA (CtS) or siRNA#10, shown with the Abcam antibody, the antibodies were ineffective in precipitating the endogenous CXXC5. Molecular masses (MM) are in kDa. IgG heavy (55 kDa) and IgG light (26 kDa) chains are indicated. (**c**) The efficiencies of antibodies to immunoprecipitate CXXC5 were also tested in transiently transfected HEK293 cells with an expression vector bearing sequences encoding a Flag-tag at the amino-terminus of CXXC5. Nuclear extracts (500 µg) were immunoprecipitated by using 5 µg of normal mouse IgG (IgG), the Flag-M2 (Sigma-Aldrich, F1804), Abcam, or CST antibody. Precipitates were then subjected to SDS-10% PAGE followed by immunoblotting with the Flag-M2 antibody. Input indicates 10% of the precipitate. Although the efficiency of the Abcam or the CST antibody to immunoprecipitate CXXC5 is lower than that of the Flag-M2 antibody, higher levels of the protein in cells allow an operational immunoprecipitation of CXXC5 by both antibodies.

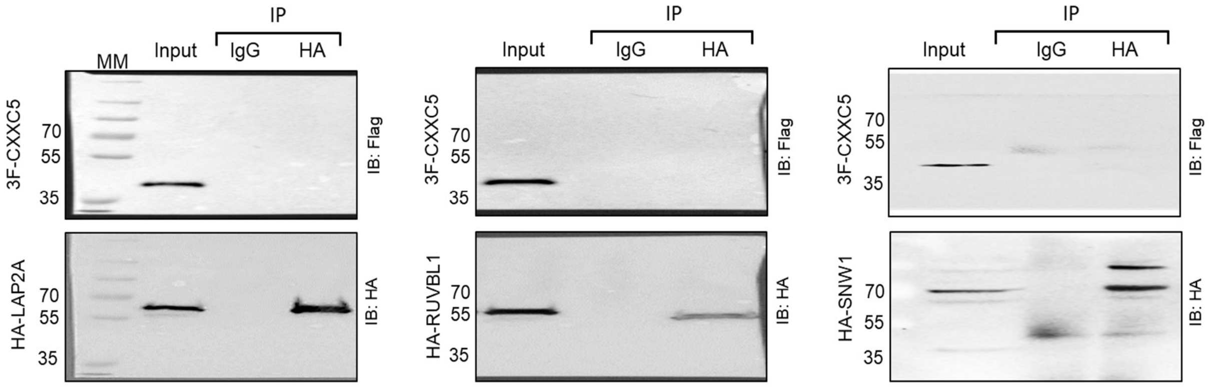

Figure S4. Initial assessment of CXXC5 interaction with LAP2A, RUVBL1, or SNW1. The nuclear extracts of HEK293 cells transiently co-transfected with the expression vectors bearing 3Flag-CXXC5 and HA-LAP2A, HA-RUVBL1, or HA-SNW1 were subjected to Co-IP using the HA antibody or the isotype-matched IgG followed by immunoblotting using the Flag or the HA antibody.

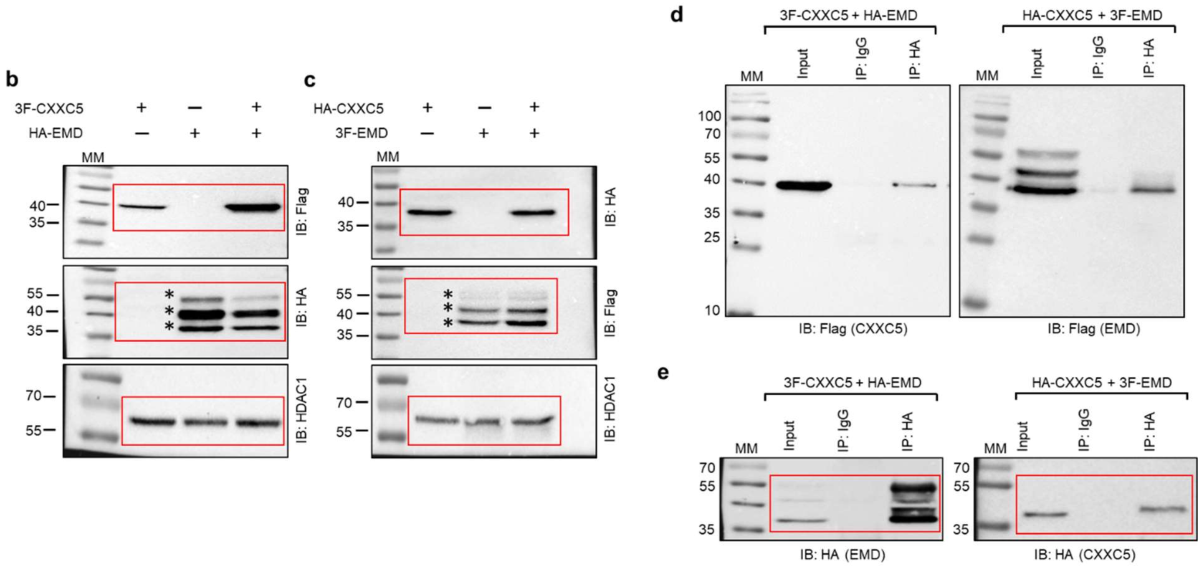

Figure S5. Interaction of EMD and CXXC5. (**b & c**) To examine the protein synthesis, HEK293 cells were transfected with the expression vector bearing (**b**) 3F-CXXC5 and/or HA-EMD cDNA; or (**c**) HA-CXXC5 and/or 3F-EMD cDNA for 48h. The synthesis of proteins was assessed by WB using the HA or the Flag antibody. HDAC1 used as a loading control was probed with the HDAC1 antibody. Star denotes distinct EMD species. (**d & e**) The nuclear extracts (500 µg) of transiently co-transfected HEK293 cells were subjected to Co-IP with the HA (d) or the isotype-matched IgG. 50 µg of nuclear lysates was used as input control. The precipitates were subjected to SDS-10%PAGE followed with WB using analyzed using the Flag (**d**) or the HA (**e**) antibody. Molecular masses (MM) in kDa are indicated. Cropped images for Fig. 2 are indicated with red boxes.

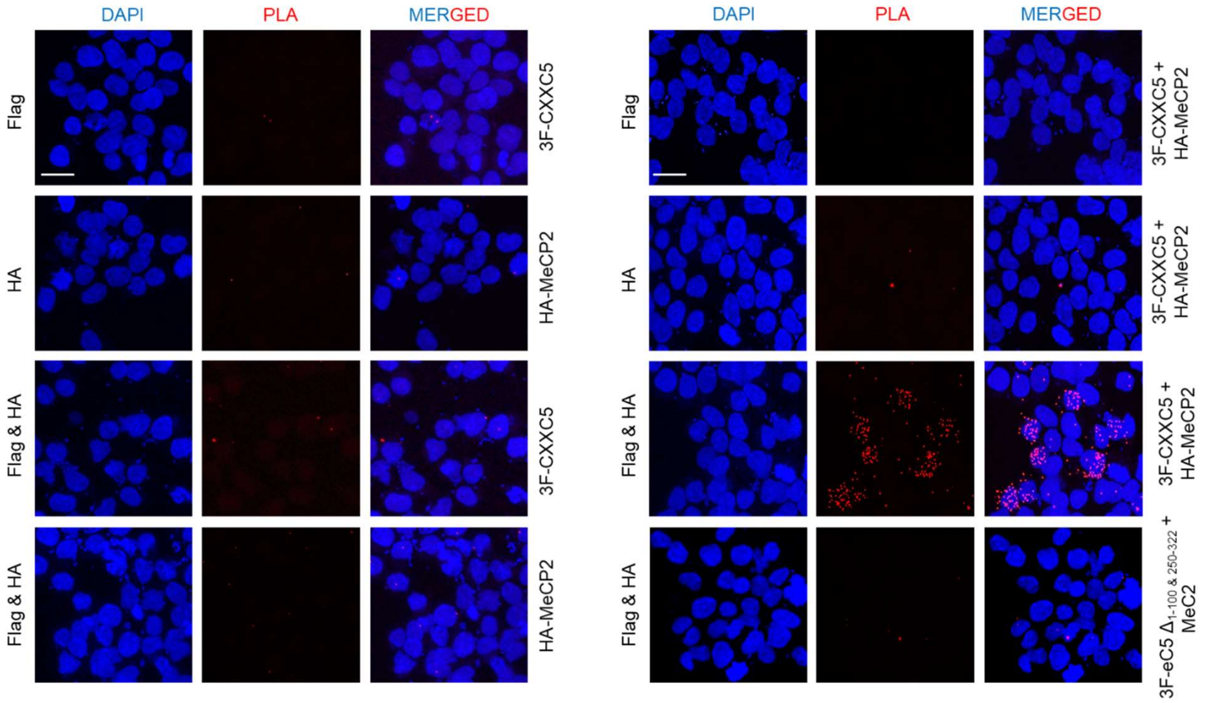

Figure S6. *In cellula* interaction of CXXC5 and MeCP2. Proximity ligation assay (PLA). To assess the *in cellula* interaction of CXXC5 and MeCP2, HEK293 cells grown in coverslips were transiently transfected the expression vector bearing the 3F-CXXC5 and/or HA-MeCP2 cDNA. Cells were also transiently co-transfected the expression vector bearing the 3F-eCXXC5$\Delta_{1\text{-}100\ \&\ 250\text{-}322}$ (3F-C5$\Delta_{1\text{-}100\ \&\ 250\text{-}322}$) and HA-MeCP2 cDNA. Cells were fixed, permeabilized, blocked, and probed with the Flag and/or the HA antibody. Cells were then subjected to fluorescent probes for circular DNA amplification. DAPI was used for nuclear staining. The scale bar is 25 μm.

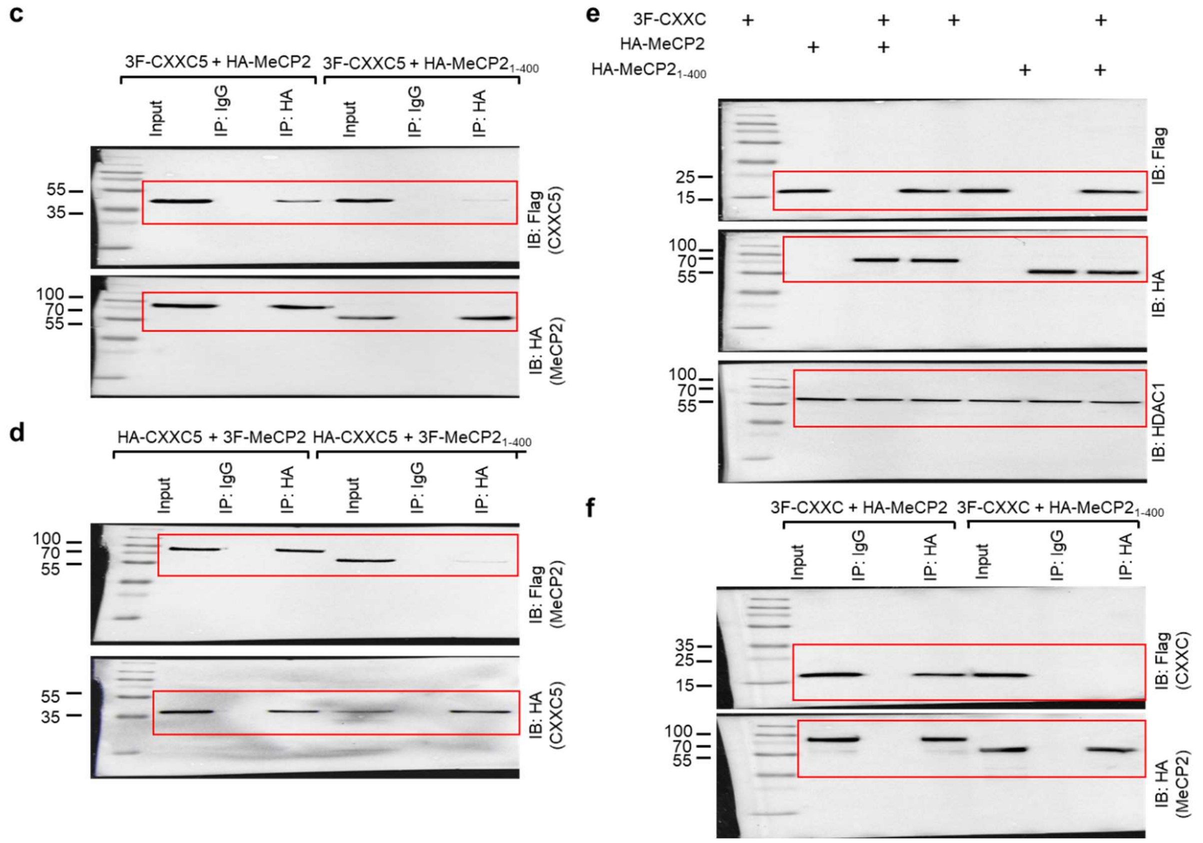

Figure S7. Identification of a sub-region of MeCP2 critical for interacting with CXXC5. (**c & d**) HEK293 cells were transiently co-transfected with the expression vector bearing cDNA for 3F-CXXC5 and HA-MeCP2 or HA-MeCP2$_{1\text{-}400}$. Nuclear extracts were subjected to Co-IP with the HA or the isotype-matched IgG. The precipitates were subjected to WB using the Flag antibody. The membrane was re-probed with the HA antibody. 10% of nuclear extracts was used as input control. Molecular masses (MM) in kDa are indicated. (**e**) Nuclear extracts of HEK293 cells transiently co-transfected with an expression vector bearing cDNA for the 3F-CXXC domain (3F-CXXC), HA-MeCP2, or HA-MeCP2$_{1\text{-}400}$, were subjected to WB using the Flag, HA or HDAC1 antibody. Molecular masses (MM) in kDa are indicated. (**f**) Nuclear extracts, 500 µg, co-synthesizing CXXC, and HA-MeCP2, or HA-MeCP2$_{1\text{-}400}$, were subjected to Co-IP with the HA antibody or the isotype-matched IgG. The precipitates were subjected to WB using the Flag antibody. The membrane was also re-probed with the HA antibody. 10% of nuclear extracts was used as input control. Molecular masses (MM) in kDa are indicated. Cropped images for Fig. 7c-f are indicated with red boxes.

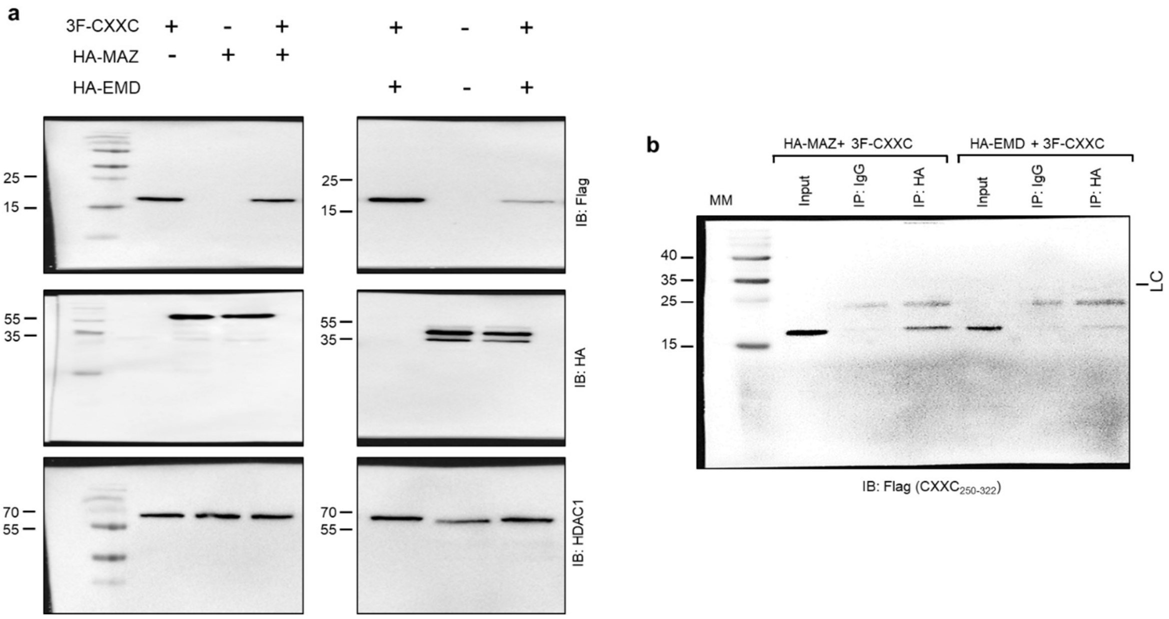

Figure S8. Interaction of the CXXC domain of CXXC5 with EMD or MAZ. To assess the interaction between the 3-Flag-CXXC domain (3F-CXXC) of CXXC5 and HA-MAZ or HA-EMD, HEK293 cells were transfected with the expression vector bearing the 3F-CXXC and/or the HA-MAZ or the HA-EMD cDNA for 48h. (**a**) Nuclear extracts (100 µg) were subjected to SDS-PAGE followed with WB using the HA or the Flag antibody. HDAC1 used as a loading control was probed with the HDAC1 antibody. (**b**) The nuclear extracts (500 µg) of transiently co-transfected HEK293 cells were subjected to Co-IP with the HA or the isotype-matched IgG. 50 µg of nuclear lysates were used as the input control. The precipitates were subjected to SDS-PAGE followed with WB using the Flag antibody. Molecular masses (MM) in kDa and the light chain of IgG (LC) are indicated.

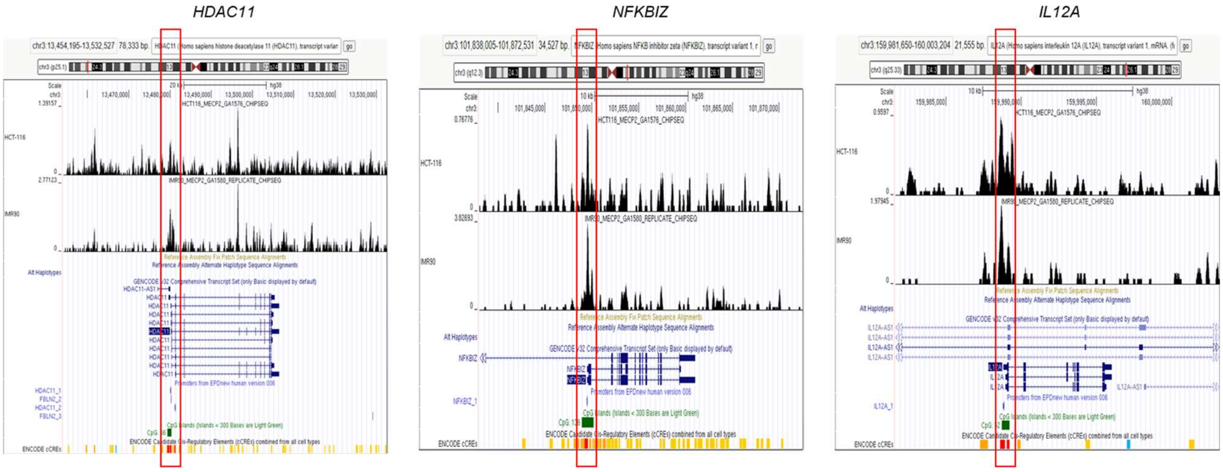

Figure S9. Binding profile of MeCP2 to the promoter, located within a CGI, of *HDAC11*, *NFKBIZ,* or *IL12A* gene. Snapshots of the binding profile of MeCP2 (Red rectangle) generated with Cistrome at gene loci using the UCSC genome browser with CGI (green), promoters (light blue arrows), and cis-regulatory elements (red boxes). To assess whether MeCP2 is enriched at the promoter region of *HDAC11*, *NFKBIZ,* or *IL12A*, datasets of IMR-90, a human lung fibroblast cell line, and HCT-166 cells derived from human colon carcinoma at the Cistrome Data Browser were visualized with the UCSC genome browser. Snapshots of the gene loci are shown. The red rectangle indicates the promoter region.

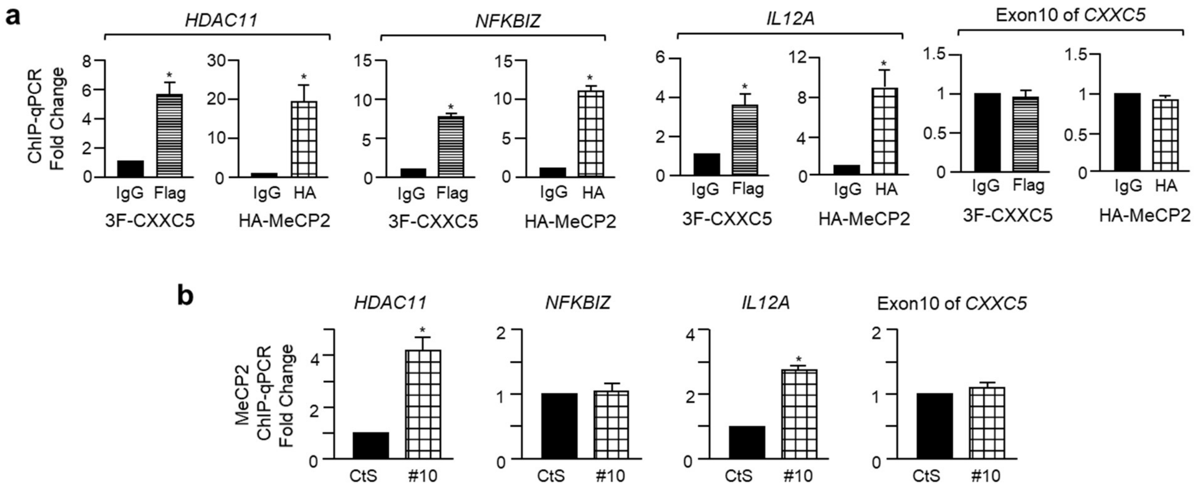

Figure S10. Assessing the interplay between CXXC5 and MeCP2 in CXXC5 target gene expressions. (**a**) To assess whether CXXC5 or MeCP2 is associated with target gene promoters, MCF7 cells were transfected with a vector bearing the 3F-CXXC5 or HA-MeCP2 cDNA for 48h. Cells were processed for ChIP using IgG, the Flag, or the HA antibody. Precipitates were subjected to qPCR using primer sets specific for target gene promoters. Results depict fold changes compared to IgG, which was set to 1. (**b**) To assess whether alterations in CXXC5 levels affect the MeCP2 loading on target promoters, MCF7 cells transfected with CtS or siRNA#10 for 48h were subjected to ChIP using IgG or a MeCP2 antibody. Recovered DNAs were subjected to qPCR using primer sets for target gene promoters. Results, normalized to IgG, depict fold changes compared to CtS, which was set to 1.

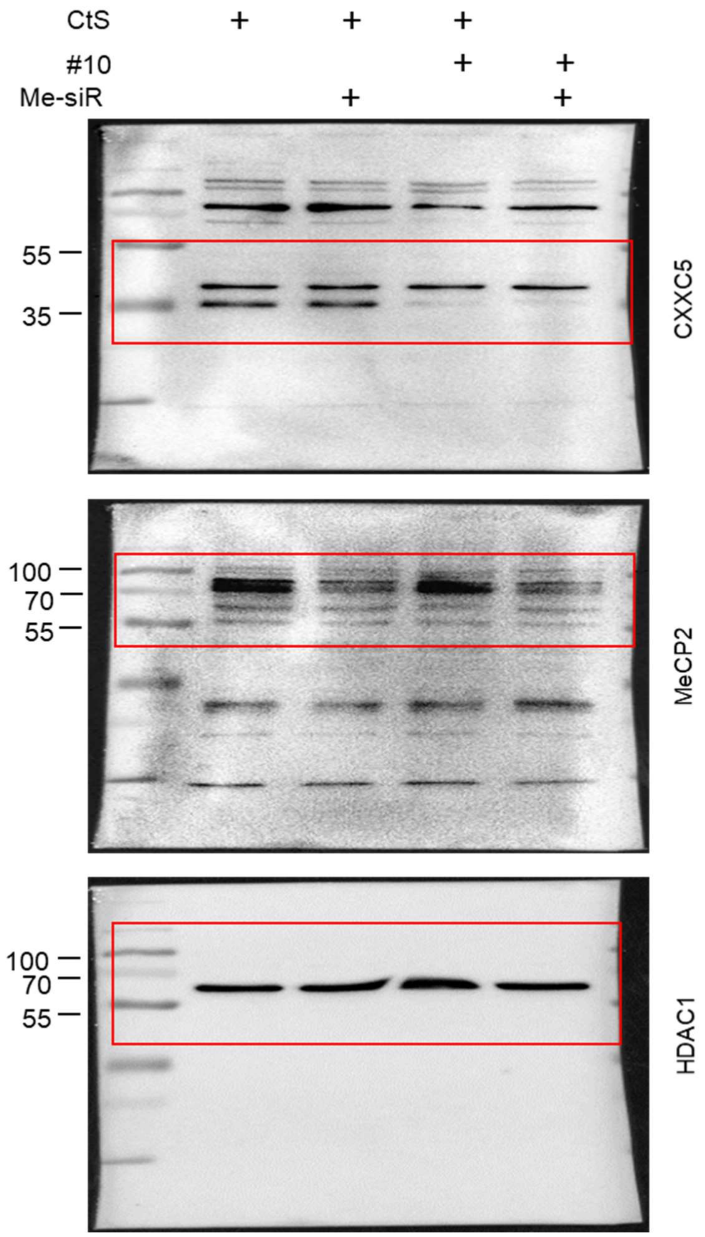

Figure S11. The effects of siRNA on protein levels of CXXC5 and/or MeCP2 in MCF7 cells. MCF7 cells were untransfected (UT), or were transiently transfected with CtS, siRNA specific for CXXC5 (#10), and/or siRNA pool for MeCP2 (Me-SiR) for 48h. To equalize the total amount of siRNA (20 nM) used in co-transfection experiments, 10 nM gene-specific siRNA was used together with 10 nM CtS. Nuclear extracts of transfected cells were subjected to WB using the CXXC5 or MeCP2 antibody. HDAC1 was probed with an HDAC1-specific antibody. MMs in kDa are indicated. (**c**) To assess the effect of reduction in CXXC5 and/or MeCP2 levels on gene expressions, MCF7 cells were transfected with CtS, #10, and/or Me-SiR as indicated for 48h. Cropped images for Fig. 8b are indicated with red boxes.

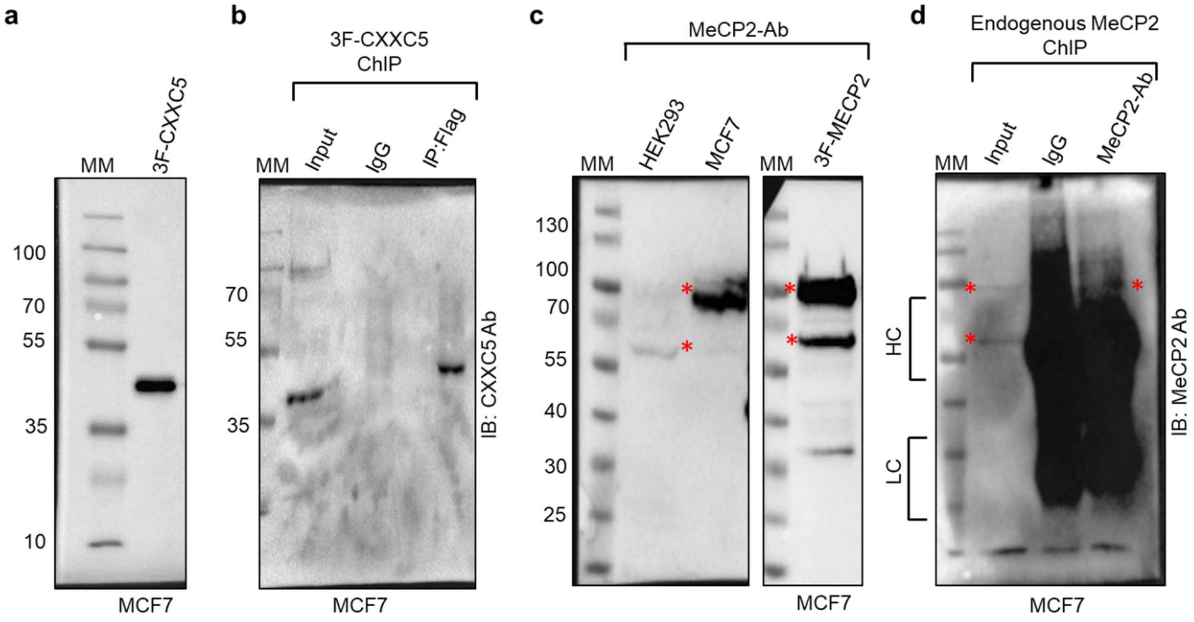

Figure S12. Immunoprecipitation of 3F-CXXC5 and ChIP of MeCP2. (**a & b**) To assess the efficiency of the Flag antibody to precipitate 3F-CXXC5 protein in ChIP assay, (**a**) **n**uclear extracts of MCF7 cells transiently transfected with an expression vector bearing the 3F-CXXC5 cDNA for 48h were subjected to SDS-10%PAGE followed with WB using an antibody specific to CXXC5. (**b**) Transfected MCF7 cells were also subjected to ChIP using the Flag antibody or the isotype-matched IgG followed by immunoblotting using the CXXC5-specific antibody. Molecular masses (MM) in kDa are indicated. (**c**) To assess the efficiency of the MeCP2 antibody to detect the endogenous MeCP2 protein in WB, the synthesis of the endogenous MeCP2 protein in HEK293 or MCF7 cells in comparison with that of 3F-MeCP2 in transiently transfected MCF7 was assessed in WB with an antibody specific to MeCP2. Molecular masses (MM) in kDa are shown. Asterisks (red) indicate MeCP2 isoforms with molecular masses of approximately 60 and 80 kDa, the latter which is likely a post-translationally modified form. (**d**) To assess the efficiency of the MeCP2 antibody to precipitate the endogenous MeCP2 protein in ChIP assay, MCF7 cells were also subjected to ChIP using the MeCP2-specific antibody or the isotype-matched IgG followed by immunoblotting using the MeCP2-specific antibody. HC and LC indicate the heavy and light chain of IgG. 5% of ChIP was used as input control.

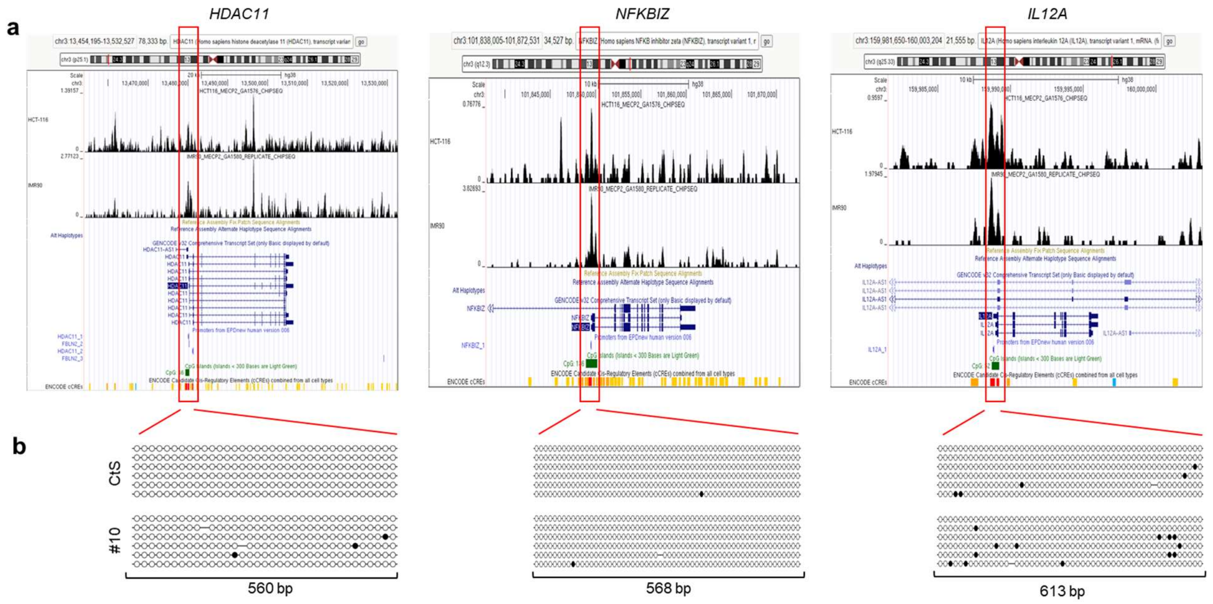

Figure S13. The methylation state of the promoter, located within a CGI, of *HDAC11*, *NFKBIZ,* or *IL12A* gene. (**a**) Snapshots of the binding profile of MeCP2 (Red rectangle) generated with Cistrome at gene loci using the UCSC genome browser with CGI (green), promoters (light blue arrows), and cis-regulatory elements (red boxes). The red rectangle indicates the promoter region. (**b**) The methylation state of promoter regions was examined with bisulfite sequencing. Isolated genomic DNA of MCF7 cells was subjected to bisulfite reaction for the conversion of unmethylated cytosine residues to uracil followed by bisulfite PCR. PCR amplicons, the lengths in base pairs (bp) of which are indicated, produced with bisulfite primers were cloned and sequenced. Aligned sequences to the corresponding regions were depicted as a lollipop distribution of two independent determinations with three independent samples each. Filled circles indicate methylated and empty circles denote unmethylated CpG dinucleotides.

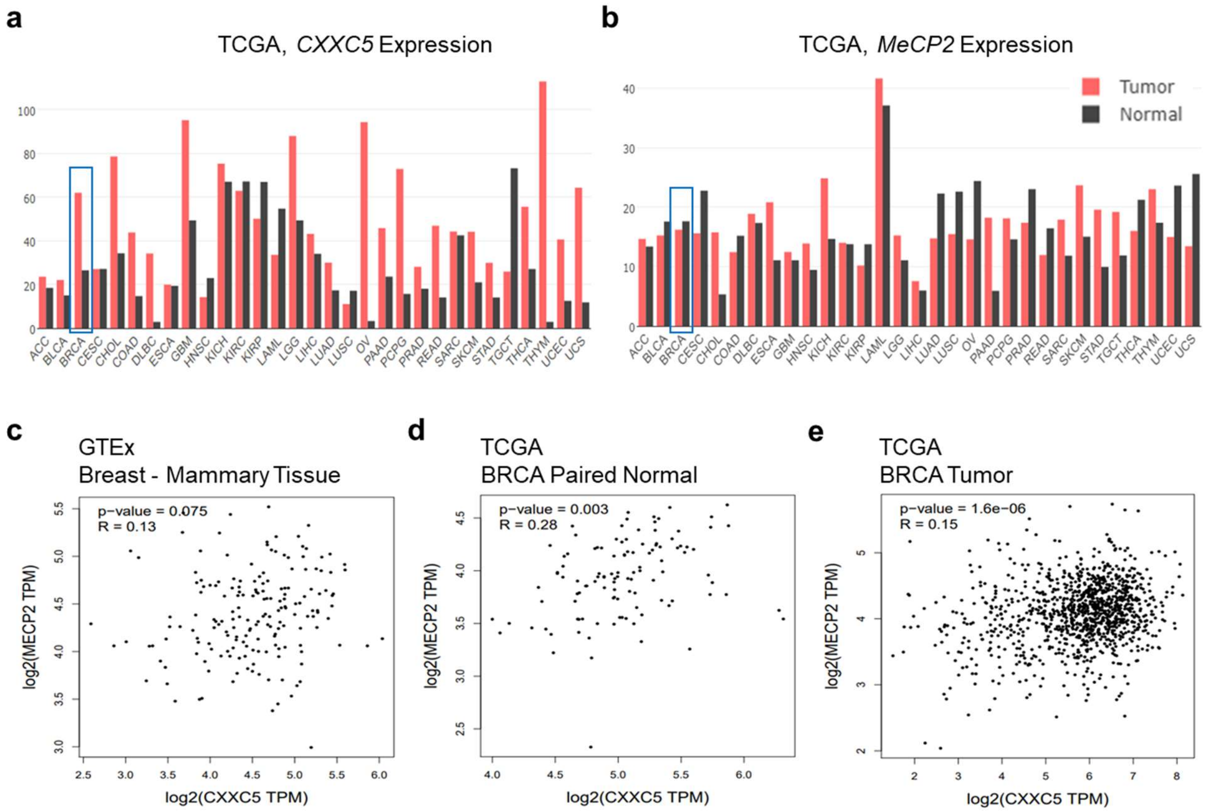

Figure S14. Expression analyses of *CXXC5* and *MeCP2* across paired normal tissues and tumor samples and the correlation between mRNA expressions of CXXC5 and MeCP2 in normal and breast tumor samples. (**a & b**) Snapshots of bar-plot analyses of the median *CXXC5* (**a**) or *MeCP2* (**b**) expression, as transcripts *per* million (TPM), in various tissue samples are shown. The red bar-plot indicates expressions in tumors and the black bar-plot denotes expressions in paired normal tissues. BRCA (blue rectangle) indicates breast invasive carcinoma. (**c-e**) Snapshots of dot-plot analyses with the use of the Pearson correlation coefficient indicate the correlation between expressions of *CXXC5* and *MeCP2* in GTEx normal breast tissue (**c**), TCGA paired normal (**d**), and TCGA breast tumor samples (**e**) as log2 TPM.

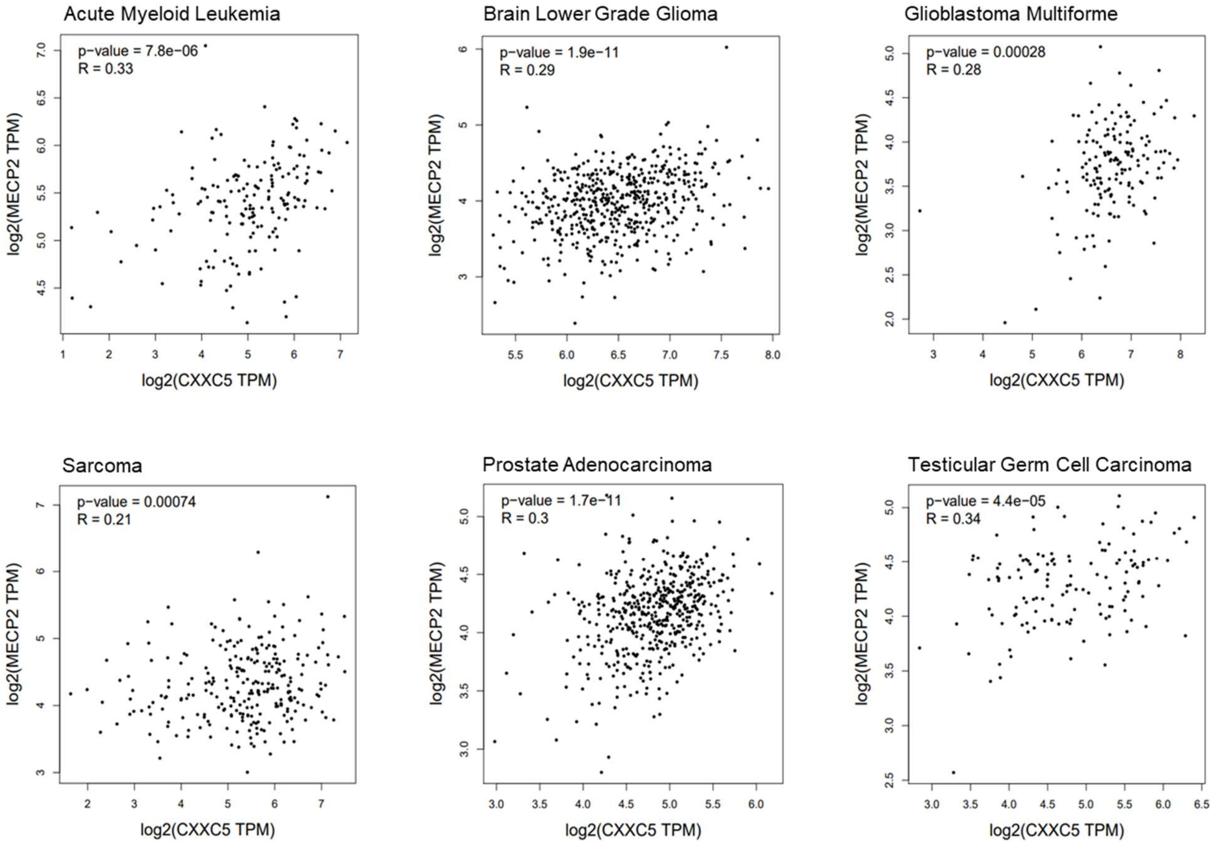

Figure S15. The correlation between mRNA expressions of *CXXC5* and *MeCP2* in tumor samples of various tissues. Snapshots of dot-plot analyses with the use of the Pearson correlation coefficient indicate the correlation between expressions of *CXXC5* and *MeCP2* in TCGA tumor samples of various tissues as log2 transcript per million (TPM) are shown.

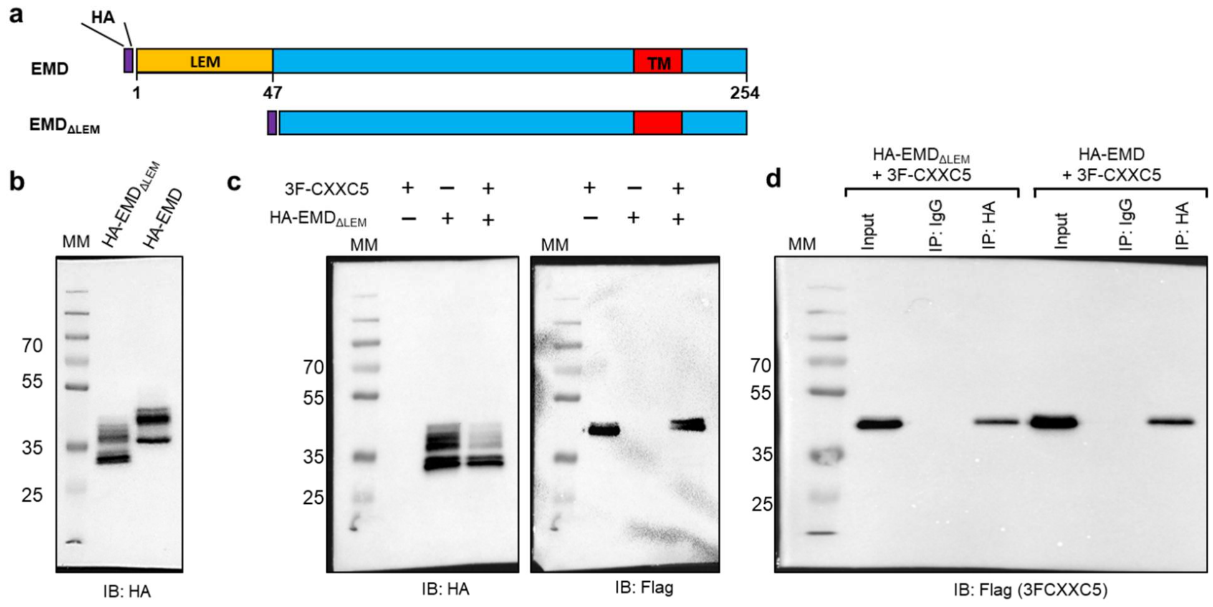

Figure S16. Interaction of EMD lacking LEM domain (EMD$_{\Delta LEM}$) with CXXC5. (a) Schematics of EMD and EMD lacking LEM domain (HA-EMD$_{\Delta LEM}$). (**b**) To assess synthesis of HA-EMD and HA-EMD$_{\Delta LEM}$, HEK293 cells were transiently transfected for 48h followed by WB using the HA antibody. **(c)** HEK293 cells were also transiently co-transfected for 48h with an expression vector (pcDNA3.1) bearing the 3F-CXXC5 and the HA-EMD$_{\Delta LEM}$ cDNAs. The synthesis of proteins (50 µg of lysate) was assessed by WB using the HA or the Flag antibody. (**d**) The nuclear extracts (500 µg) of HEK293 cells co-synthesizing 3xF-CXXC5 and HA-EMD$_{\Delta LEM}$ or 3xF-CXXC5 and HA-EMD were subjected to Co-IP with the HA or the isotype-matched IgG. 10% of nuclear lysates was used as input control. The precipitates were subjected to SDS-10%PAGE followed with WB using analyzed using the Flag antibody. Molecular masses (MM) in kDa are indicated.

TABLE S1. CXXC5 Proximity Interactors, G AYAZ *et al.*

No	Accession #	String Id	Preferred Name	Description	# AAs	MW [kDa]	calc. pI	Biological Replicate I										Biological Replicate II									
								1st Technical Replicate					2nd Technical Replicate					1st Technical Replicate					2nd Technical Replicate				
No	Accession #	String Id	Preferred Name	Description	# AAs	MW [kDa]	calc. pI	Σcoverage	Σ# Proteins	Σ# Unique Peptides	Σ# Peptides	Σ# PSMs	Σcoverage	Σ# Proteins	Σ# Unique Peptides	Σ# Peptides	Σ# PSMs	Σcoverage	Σ# Proteins	Σ# Unique Peptides	Σ# Peptides	Σ# PSMs	Σcoverage	Σ# Proteins	Σ# Unique Peptides	Σ# Peptides	Σ# PSMs
1	Q9H2P0	9606.ENSP00000379346	ADNP	Activity-dependent neuroprotector homeobox protein	1102	123.49	7.34	15.06	2	14	14	25	18.87	2	15	15	26	6.81	2	5	5	10	6.81	2	5	5	10
2	P05549	9606.ENSP00000368924	TFAP2A	Transcription factor AP-2-alpha	437	48.03	8.02	24.49	14	5	14	33	26.54	14	8	16	38	11.44	13	3	4	7	11.44	15	4	4	10
3	Q8WWM7	9606.ENSP00000378917	ATXN2L	Ataxin-2-like protein	1075	113.30	8.59	2.79	9	2	2	4	2.14	8	1	1	2	2.14	8	1	1	2	2.14	8	1	1	2
4	Q9NRL2	9606.ENSP00000353458	BAZ1A	Bromodomain adjacent to zinc finger domain protein 1A	1556	178.59	6.60	1.99	2	2	2	5	4.05	2	3	3	8	5.14	3	6	6	12	2.76	3	4	4	8
5	Q9UIG0	9606.ENSP00000342434	BAZ1B	Tyrosine-protein kinase BAZ1B	1483	170.80	8.48	18.81	2	25	25	49	19.82	2	26	26	53	18.88	2	20	20	42	16.93	2	17	17	36
6	Q9NYF8	9606.ENSP00000435210	BCLAF1	Bcl-2-associated transcription factor 1	920	106.06	9.98	8.15	10	6	6	10	4.57	9	3	3	4	5.33	10	3	3	8	5.22	9	3	3	8
7	Q12830	9606.ENSP00000307208	BPTF	Nucleosome-remodeling factor subunit BPTF	3046	338.05	6.54	3.05	5	8	8	13	3.61	6	9	9	17	1.74	5	4	4	8	2.43	6	5	5	10
8	Q8N163	9606.ENSP00000310670	CCAR2	Cell cycle and apoptosis regulator protein 2	923	102.84	5.22	1.52	3	1	1	2	1.52	3	1	1	2	1.52	3	1	1	2	1.52	3	1	1	2
9	Q14839	9606.ENSP00000349508	CHD4	Chromodomain-helicase-DNA-binding protein 4	1912	217.87	5.86	9.36	3	11	14	28	12.45	3	15	19	39	10.41	6	12	16	33	10.3	4	13	17	35
10	Q9HCK8	9606.ENSP00000382863	CHD8	Chromodomain-helicase-DNA-binding protein 8	2581	290.34	6.47	7.25	7	12	13	24	8.21	6	12	14	28	5.85	4	9	9	18	4.49	4	7	7	14
11	Q9BR76	9606.ENSP00000377471	CORO1B	Coronin-1B	489	54.20	5.88	15.13	4	5	5	10	10.43	4	3	3	6	7.77	4	3	3	6	5.52	4	2	2	6
12	Q16630	9606.ENSP00000266679	CPSF6	Cleavage and polyadenylation specificity factor subunit 6	551	59.17	7.15	2.72	4	1	1	2	9.44	4	3	3	6	2.54	4	1	1	2	7.08	4	2	2	4
13	P49711	9606.ENSP00000264010	CTCF	Transcriptional repressor CTCF	727	82.73	6.96	8.39	2	4	4	9	10.73	2	5	5	9	8.1	4	4	4	9	11.2	5	5	5	9
14	P39880	9606.ENSP00000353401	CUX1	Homeobox protein cut-like 1	1505	164.09	5.90	16.68	11	18	18	33	14.68	11	16	16	32	5.05	11	6	6	12	4.98	11	5	5	10
**15**	**Q7LFL8**	**9606.ENSP00000302543**	**CXXC5**	**CXXC-type zinc finger protein 5**	**322**	**32.96**	**9.10**	**45.03**	**12**	**22**	**22**	**108**	**45.03**	**12**	**20**	**20**	**116**	**31.37**	**12**	**8**	**8**	**50**	**31.37**	**12**	**8**	**8**	**59**
16	Q9NR30	9606.ENSP00000346120	DDX21	Nucleolar RNA helicase 2	783	87.29	9.28	5.75	2	4	4	6	5.49	2	3	3	5	2.81	2	1	1	2	2.81	2	1	1	2
17	P17844	9606.ENSP00000225792	DDX5	Probable ATP-dependent RNA helicase DDX5	614	69.10	8.92	16.29	14	9	9	17	19.87	14	11	11	20	8.63	9	4	4	8	8.63	9	4	4	8
18	Q08211	9606.ENSP00000356520	DHX9	ATP-dependent RNA helicase A	1270	140.87	6.84	5.75	2	6	6	10	4.41	1	6	6	9	5.51	2	5	5	10	4.09	2	4	4	8
19	Q9BTC0	9606.ENSP00000266070	DIDO1	Death-inducer obliterator 1	2240	243.72	7.88	1.38	2	2	2	3	2.5	4	4	4	8	3.75	2	7	7	13	3.93	2	6	6	14
20	O60832	9606.ENSP00000358563	DKC1	H/ACA ribonucleoprotein complex subunit 4	514	57.64	9.42	10.31	6	3	3	6	6.61	6	3	3	4	2.53	5	1	1	2	2.53	5	1	1	2
21	P32519	9606.ENSP00000239882	ELF1	ETS-related transcription factor Elf-1	619	67.46	5.21	17.61	11	7	7	15	9.69	6	3	4	8	5.82	2	2	2	4	2.91	2	1	1	2
22	Q6PJG2	9606.ENSP00000286523	ELMSAN1	ELM2 and SANT domain-containing protein 1	1045	114.92	9.19	5.17	2	3	3	6	6.6	3	4	4	8	1.82	1	2	2	4	2.87	1	3	3	6
23	Q8WYP5	9606.ENSP00000355465	ELYS	Protein ELYS	2266	252.34	6.60	3.18	3	4	4	7	2.43	3	3	3	6	1.37	3	2	2	3	0.71	3	1	1	2
24	P50402	9606.ENSP00000358857	EMD	Emerin	254	28.98	5.50	10.24	2	2	2	3	10.24	2	2	2	3	12.24	3	2	2	3	14.1	4	2	2	4
25	Q7Z589	9606.ENSP00000433205	C11orf30 (EMSY)	Protein EMSY	1322	141.38	9.33	6.43	12	5	5	11	14.07	13	11	11	17	4.54	9	3	3	5	2.04	8	2	2	4
26	P22087	9606.ENSP00000221801	FBL	rRNA 2'-methyltransferase fibrillarin	321	33.76	10.18	18.38	10	4	4	8	24.92	10	6	6	12	22.43	10	5	5	10	12.77	10	3	3	6
27	Q6ISB3	9606.ENSP00000251808	GRHL2	Grainyhead-like protein 2 homolog	625	71.06	6.44	19.04	2	7	7	16	23.84	2	9	9	22	15.04	2	6	6	11	17.6	2	7	7	12
28	P78347	9606.ENSP00000460070	GTF2I	General transcription factor II-I	998	112.35	6.39	2.51	4	2	2	4	1.5	4	1	1	2	1.6	4	1	1	3	2.91	5	2	2	6
29	O60814	9606.ENSP00000349430	HIST1H2BK	Histone H2B type 1-K	126	13.88	10.32	69.84	2	8	13	50	74.6	3	1	16	53	19.05	17	2	2	14	19.05	17	2	2	12
30	P51610	9606.ENSP00000309555	HCFC1	Host cell factor 1	2035	208.60	7.46	7.27	5	12	12	25	7.52	5	12	12	22	5.45	5	8	8	18	6.54	5	10	10	20
31	Q9UGU5	9606.ENSP00000216106	HMGXB4	HMG domain-containing protein 4	601	65.67	9.32	6.16	3	2	2	3	6.16	3	3	3	5	2.83	3	1	1	2	2.83	3	1	1	2
32	O43390	9606.ENSP00000363745	HNRNPR	Heterogeneous nuclear ribonucleoprotein R	633	70.90	8.13	7.9	11	3	3	6	9.64	6	4	5	10	7.74	5	2	5	9	4.27	5	1	3	6
33	P08107	9606.ENSP00000364802	HSP71(HSPA1A)	Heat shock 70 kDa protein 1A/1B	641	70.01	5.66	8.74	9	3	4	10	9.2	10	3	4	6	2.5	3	1	1	4	6.55	4	3	3	5
34	P11142	9606.ENSP00000432083	HSPA8	Heat shock cognate 71 kDa protein	646	70.85	5.52	8.82	16	4	5	10	4.49	15	2	3	6	4.49	12	2	2	4	4.18	13	2	2	4
35	P55010	9606.ENSP00000216554	IF5 (EIF5)	Eukaryotic translation initiation factor 5	431	49.19	5.58	12.06	3	5	5	8	12.53	1	6	6	10	16.01	7	6	6	11	6.96	7	3	3	6
36	P08727	9606.ENSP00000355124	KRT19	Keratin, type I cytoskeletal 19	400	44.08	5.14	11.5	15	2	5	10	19.75	17	4	7	13	25.75	9	3	12	28	21.5	8	2	12	24
37	Q9Y2K7	9606.ENSP00000432786	KDM2A	Lysine-specific demethylase 2A	1162	132.71	7.58	1.46	5	1	1	2	2.5	5	2	2	4	1.46	5	1	1	2	1.46	5	1	1	2
38	P46013	9606.ENSP00000357643	KI67 (MKI67)	Antigen KI-67	3256	358.47	9.45	13.85	3	29	29	55	17.57	3	35	35	66	11.92	3	24	24	50	12.07	3	26	26	50
39	Q03164	9606.ENSP00000436786	KMT2A	Histone-lysine N-methyltransferase 2A	3969	431.50	9.09	3.2	4	9	9	17	3.63	4	11	11	21	6.35	5	16	16	30	5.29	5	14	14	27
40	Q9UMN6	9606.ENSP00000398837	KMT2B	Histone-lysine N-methyltransferase 2B	2715	293.33	8.22	0.88	2	2	2	4	0.99	2	2	2	4	1.18	1	2	2	4	1.18	1	2	2	4
41	P42166	9606.ENSP00000266732	LAP2A (TMPO)	Lamina-associated polypeptide 2, isoform alpha	694	75.45	7.66	43.37	2	18	25	46	41.07	2	19	26	53	29.68	2	10	15	29	19.16	3	6	11	21
42	P42167	9606.ENSP00000266732	LAP2B (TMPO)	Lamina-associated polypeptide 2, isoforms beta/gamma	454	50.64	9.38	37.22	5	9	16	35	46.92	5	10	17	34	26.65	5	3	8	15	23.79	5	2	7	15
43	Q6PKG0	9606.ENSP00000336721	LARP1	La-related protein 1	1096	123.43	8.82	2.19	4	1	1	2	1.19	4	1	1	2	2.65	5	2	2	4	2.65	5	2	2	3
44	Q6MZP7	9606.ENSP00000341947	LIN54	Protein lin-54 homolog	749	79.44	9.01	8.54	5	4	4	8	6.81	4	3	3	6	4.94	4	3	3	6	4.94	4	3	3	6
45	Q96AG4	9606.ENSP00000225972	LRRC59	Leucine-rich repeat-containing protein 59	307	34.91	9.57	18.24	1	4	4	7	13.36	1	4	4	7	5.21	1	1	1	2	8.14	1	2	2	4
46	Q5T3J3	9606.ENSP00000358778	LRIF1	Ligand-dependent nuclear receptor-interacting factor 1	769	84.52	9.72	13.26	1	6	6	10	12.74	1	5	5	7	7.8	1	4	4	8	7.67	1	4	4	8
47	Q86UE4	9606.ENSP00000338235	MTDH	Protein LYRIC	582	63.80	9.32	3.44	2	1	1	2	3.26	1	1	1	1	1.89	3	1	1	2	1.89	3	1	1	2
48	P56270	9606.ENSP00000219782	MAZ	Myc-associated zinc finger protein	477	48.58	8.95	6.08	5	1	2	4	8.6	5	2	3	6	6.18	6	1	2	4	9.6	6	2	3	6
49	Q14676	9606.ENSP00000365588	MDC1	Mediator of DNA damage checkpoint protein 1	2089	226.53	5.47	2.25	5	3	3	6	1.63	5	2	2	4	3.06	4	3	3	6	3.88	4	4	4	8
50	P51608	9606.ENSP00000395535	MECP2	Methyl-CpG-binding protein 2	486	52.41	9.95	3.29	4	1	1	2	7.41	4	2	2	3	4.94	4	2	2	4	8.23	4	4	4	8
51	Q9ULH7	9606.ENSP00000339086	MKL2	MKL/myocardin-like protein 2	1088	118.05	6.28	10.94	6	6	6	11	10.66	16	7	7	12	3.49	3	2	2	4	2.21	6	2	2	4
52	Q9H0A0	9606.ENSP00000257829	NAT10	N-acetyltransferase 10	1025	115.66	8.27	2.93	3	2	2	3	5.46	5	4	4	7	2.54	3	2	2	4	1.66	3	1	1	2
53	O00712	9606.ENSP00000370340	NFIB	Nuclear factor 1 B-type	420	47.41	8.87	2.62	6	1	1	2	8.1	8	2	2	4	3.33	10	1	1	3	3.33	10	1	1	2
54	Q14938	9606.ENSP00000380781	NFIX	Nuclear factor 1 X-type	502	55.06	8.68	2.39	8	1	1	2	2.39	8	1	1	2	2.79	12	1	1	4	2.79	12	1	1	4
55	Q6P4R8	9606.ENSP00000436926	NFRKB	Nuclear factor related to kappa-B-binding protein	1299	138.92	9.25	7.54	4	8	8	14	6	4	6	6	12	3.93	3	3	3	6	2.31	3	2	2	4
56	Q15233	9606.ENSP00000276079	NONO	Non-PU domain-containing octamer-binding protein	471	54.20	8.95	7.43	5	3	3	6	2.55	2	1	1	2	6.37	4	2	2	4	3.18	4	1	1	2
57	P49116	9606.ENSP00000483059	NR2C2	Nuclear receptor subfamily 2 group C member 2	596	65.37	6.28	2.52	5	1	1	2	2.52	5	1	1	2	4.87	3	2	2	4	4.87	3	2	2	4
58	O96028	9606.ENSP00000372351	NSD2	Histone-lysine N-methyltransferase NSD2	1365	152.16	8.69	1.47	7	1	1	2	2.78	7	2	2	3	1.17	4	1	1	2	1.98	4	2	2	3
59	Q14980-2	9606.ENSP00000377298	NUMA1	Nuclear mitotic apparatus protein 1	2101	236.37	5.80	21.13	9	44	44	60	21.56	9	41	41	55	31.22	22	54	54	62	30.75	22	51	51	60
60	P19338	9606.ENSP00000318195	NCL	Nucleolin	710	76.57	4.70	27.04	6	12	12	23	23.66	6	10	10	24	13.38	2	6	6	14	15.35	2	7	7	16
61	C9JPD0	9606.ENSP00000384551	NXPH1	Neurexophilin-1 (Fragment)	143	16.46	9.35	7.69	4	1	1	3	7.69	4	1	1	1	7.69	4	1	1	1	7.69	4	1	1	2
62	Q13416	9606.ENSP00000234296	ORC2	Origin recognition complex subunit 2	577	65.93	6.51	4.33	1	2	2	4	1.73	1	1	1	1	3.12	1	1	1	2	3.12	1	1	1	2
63	Q86YP4	9606.ENSP00000353463	GATAD2A (P66A)	Transcriptional repressor p66-alpha	633	68.02	9.94	30.02	11	16	17	39	33.97	11	17	18	43	14.06	11	5	6	11	12.48	11	5	5	10
64	Q8WXI9	9606.ENSP00000357644	GATAD2B (P66B)	Transcriptional repressor p66-beta	593	65.22	9.70	21.92	1	8	9	21	18.04	1	6	7	18	16.86	1	5	6	12	11.64	1	4	4	7
65	Q8NC51	9606.ENSP00000360034	SERBP1	Plasminogen activator inhibitor 1 RNA-binding protein	408	44.94	8.65	7.11	4	3	3	4	11.76	4	4	4	6	4.41	4	1	1	2	4.41	4	1	1	2
66	Q15365	9606.ENSP00000305556	PCBP1	Poly(rC)-binding protein 1	356	37.47	7.09	14.61	2	1	3	6	12.92	2	3	4	7	9.55	20	2	2	4	9.55	20	2	2	4
67	Q8WWQ0	9606.ENSP00000275034	PHIP	PH-interacting protein	1821	206.56	8.85	4.01	1	4	4	6	3.02	1	3	3	5	4.06	6	5	5	10	4.17	6	5	5	10
68	P14923	9606.ENSP00000377508	PLAK (JUP)	Junction plakoglobin	745	81.69	6.14	2.28	1	1	1	2	2.42	1	1	1	2	6.31	5	3	3	6	8.59	5	5	5	10
69	Q7Z3K3	9606.ENSP00000271715	POGZ	Pogo transposable element with ZNF domain	1410	155.24	7.40	1.42	7	2	2	4	2.2	6	3	3	6	2.06	6	2	2	4	2.06	6	2	2	4
70	Q96QC0	9606.ENSP00000365694	PPP1R10	Serine/threonine-protein phosphatase 1 regulatory subunit 10	940	99.00	9.17	11.38	1	8	8	12	13.09	1	9	9	14	5.43	1	3	3	5	5.21	1	3	3	6
71	O43395	9606.ENSP00000315379	PRPF3	U4/U6 small nuclear ribonucleoprotein Prp3	683	77.48	9.50	17.72	2	11	11	20	21.82	2	12	12	19	12.3	2	6	6	11	13.03	2	6	6	13
72	P35251	9606.ENSP00000371321	RFC1	Replication factor C subunit 1	1148	128.18	9.36	1.05	3	1	1	2	1.05	3	1	1	2	1.83	3	2	2	3	1.05	3	1	1	2
73	P22670	9606.ENSP00000254325	RFX1 (HGNC:9982)	MHC class II regulatory factor RFX1	979	104.69	6.29	4.19	1	3	3	6	7.05	1	4	4	9	1.33	1	1	1	2	1.33	1	1	1	2
74	Q02543	9606.ENSP00000222247	RPL18A	60S ribosomal protein L18a	176	20.75	10.71	19.89	5	3	3	7	14.2	5	3	3	5	7.39	5	1	1	2	7.39	5	1	1	2
75	P22626	9606.ENSP00000346694	ROA2 (HNRNPA2B1	Heterogeneous nuclear ribonucleoproteins A2/B1	353	37.41	8.95	18.7	3	4	4	8	22.38	3	6	6	11	16.43	3	1	4	9	15.01	3	2	4	10
76	Q92766	9606.ENSP00000369270	RREB1	Ras-responsive element-binding protein 1	1687	181.31	6.98	6.4	8	8	8	12	5.99	9	8	8	13	1.54	5	2	2	4	2.73	5	3	3	6
77	P62851	9606.ENSP00000435096	RPS25	40S ribosomal protein S25	125	13.73	10.11	15.2	1	2	2	4	15.2	1	2	2	4	24	1	3	3	6	24	1	3	3	6
78	Q9Y265	9606.ENSP00000318297	RUVBL1	RuvB-like 1	456	50.20	6.42	12.28	3	5	5	7	12.28	3	5	5	8	12.28	5	4	4	7	12.28	5	4	4	8
79	P31151	9606.ENSP00000357712	S100A7	Protein S100-A7	101	11.46	6.77	13.86	1	1	1	2	10.89	2	1	1	1	10.89	2	1	1	2	10.89	2	1	1	2
80	Q9UQR0	9606.ENSP00000251900	SCML2	Sex comb on midleg-like protein 2	700	77.21	8.54	13.57	3	6	6	13	15.71	3	7	7	15	6.86	3	3	3	8	9	3	4	4	8
81	Q13435	9606.ENSP00000318861	SF3B2	Splicing factor 3B subunit 2	895	100.16	5.67	0.78	3	1	1	2	3.69	4	3	3	6	1.45	4	1	1	2	1.45	4	1	1	2
82	O60264	9606.ENSP00000283131	SMARCA5	SWI/SNF-related matrix-associated actin-dependent regulator of chromatin subfamily A member 5	1052	121.83	8.09	17.68	3	15	15	34	13.5	3	12	12	26	5.32	3	5	5	10	4.37	3	5	5	10
83	A6NHR9	9606.ENSP00000326603	SMCHD1	Structural maintenance of chromosomes flexible hinge domain-containing protein 1	2005	226.23	7.30	2	4	3	3	6	3.14	4	5	5	9	1.75	4	3	3	6	1.75	4	3	3	6
84	Q13573	9606.ENSP00000261531	SNW1	SNW domain-containing protein 1	536	61.46	9.52	17.54	4	7	7	15	19.22	4	7	7	12	9.33	3	4	4	7	7.28	4	2	2	4
85	G5E9C0	9606.ENSP00000258381	SP110	SP110 nuclear body protein, isoform CRA_b	547	61.70	9.54	25.23	10	1	13	18	28.15	10	1	16	22	34.37	11	17	17	25	34.92	11	19	19	27
86	Q8IYB3	9606.ENSP00000326261	SRRM1	Serine/arginine repetitive matrix protein 1	904	102.27	11.84	7.63	5	4	4	7	5.53	5	3	3	5	8.19	6	6	6	14	10.95	6	7	7	14
87	P42224	9606.ENSP00000354394	STAT1	Signal transducer and activator of transcription 1-alpha/beta	750	87.28	6.05	1.6	3	1	1	2	1.6	3	1	1	2	3.47	4	2	2	4	3.33	3	2	2	4
88	Q13148	9606.ENSP00000240185	TARDBP	TAR DNA-binding protein 43	414	44.71	6.19	4.35	3	1	1	2	4.35	3	1	1	2	2.9	16	1	1	2	2.9	16	1	1	2
89	P37802	9606.ENSP00000357076	TAGLN2	Transgelin-2	199	22.38	8.25	13.07	5	2	2	4	13.07	5	2	2	4	26.13	3	3	3	6	26.13	3	3	3	6
90	Q9UGU0	9606.ENSP00000352463	TCF20	Transcription factor 20	1960	211.64	9.04	3.52	2	4	4	6	2.45	2	3	3	6	2.24	2	2	3	6	1.22	2	1	1	2
91	P50990	9606.ENSP00000286788	TCPQ (CCT8)	T-complex protein 1 subunit theta	548	59.58	5.60	11.68	5	6	6	11	12.77	4	5	5	9	14.78	4	6	6	12	14.78	4	6	6	14
92	Q5QJE6	9606.ENSP00000411010	TDIF2 (DNTTIP2)	Deoxynucleotidyltransferase terminal-interacting protein 2	756	84.42	6.16	4.1	1	2	2	4	3.84	2	3	3	6	3.84	2	2	2	4	3.84	2	2	2	4
93	P28347	9606.ENSP00000435233	TEAD1	Transcriptional enhancer factor TEF-1	426	47.91	8.15	2.35	13	1	1	2	2.82	14	1	1	2	2.58	14	1	1	2	2.58	14	1	1	2
94	Q86V81	9606.ENSP00000421592	THOC4 (ALYREF)	TH complex subunit 4	257	26.87	11.15	20.23	2	3	3	4	20.23	2	3	3	5	4.28	2	1	1	2	4.28	2	1	1	4
95	Q13263	9606.ENSP00000253024	TRIM28	Transcription intermediary factor 1-beta	835	88.49	5.77	6.83	3	4	4	8	2.87	3	2	2	6	4.31	3	2	2	4	4.31	3	2	2	4
96	P11388	9606.ENSP00000411532	TOP2A	DNA topoisomerase 2-alpha	1531	174.28	8.72	4.25	4	5	5	9	6.4	5	8	8	11	4.77	4	4	5	8	1.7	4	1	2	4
97	Q02880	9606.ENSP00000396704	TOP2B	DNA topoisomerase 2-beta	1626	183.15	8.00	0.55	3	1	1	2	1.29	3	2	2	4	3.01	3	3	4	8	3.01	3	3	4	8
98	O94842	9606.ENSP00000477868	TOX4	TX high mobility group box family member 4	621	66.15	5.06	16.43	10	8	8	17	14.98	10	9	9	20	10.14	10	4	4	7	5.64	9	3	3	4
99	P12270	9606.ENSP00000356448	TPR	Nucleoprotein TPR	2363	267.13	5.02	0.59	1	1	1	2	1.35	1	2	2	5	2.2	1	4	4	9	4.06	1	7	7	15
100	Q14258	9606.ENSP00000323889	TRIM25	E3 ubiquitin/ISG15 ligase TRIM25	630	70.93	8.09	1.6	1	1	1	3	1.9	1	1	1	2	1.9	1	1	1	2	1.9	1	1	1	1
101	Q9UPN9	9606.ENSP00000351250	TRIM33	E3 ubiquitin-protein ligase TRIM33	1127	122.46	6.67	5.68	4	5	5	7	6.03	4	5	5	9	2.13	4	2	2	4	2.13	4	2	2	4
102	Q9UHF7	9606.ENSP00000379065	TRPS1	Zinc finger transcription factor Trps1	1281	141.43	7.58	11.94	7	11	11	21	12.18	7	12	12	25	2.97	7	3	3	6	3.51	6	3	3	5
103	Q14157	9606.ENSP00000389445	UBAP2L	Ubiquitin-associated protein 2-like	1087	114.47	7.11	1.84	11	2	2	4	3.13	11	3	3	5	4.05	11	3	3	6	5.7	11	4	4	8
104	Q9Y4E8	9606.ENSP00000280377	USP15	Ubiquitin carboxyl-terminal hydrolase 15	981	112.35	5.22	1.6	4	1	1	2	1.8	3	1	1	3	1.43	3	1	1	2	1.43	3	1	1	2
105	P12956	9606.ENSP00000352257	XRCC6	X-ray repair cross-complementing protein 6	609	69.80	6.64	14.12	3	7	7	16	20.69	3	10	10	18	8.37	3	4	4	8	10.67	3	5	5	14
106	Q96KM6	9606.ENSP00000393795	ZNF512B	Zinc finger protein 512B	892	97.20	9.83	13	1	7	7	12	8.63	1	5	5	12	5.94	1	3	3	5	5.94	1	3	3	5
107	Q96C00	9606.ENSP00000378503	ZBTB9	Zinc finger and BTB domain-containing protein 9	473	50.57	6.76	4.02	3	1	1	2	5.92	3	2	2	3	4.02	3	1	1	2	4.02	3	1	1	2
108	Q9UQR1	9606.ENSP00000353863	ZNF148	Zinc finger protein 148	794	88.92	6.48	7.05	1	3	4	7	9.32	1	4	5	8	5.54	1	3	3	6	3.78	1	2	2	4
109	Q14966	9606.ENSP00000386433	ZNF638	Zinc finger protein 638	1978	220.49	6.38	0.86	4	1	1	2	0.86	4	1	1	2	1.47	4	2	2	4	0.86	4	1	1	2

TABLE S2. Primers, G AYAZ *et al.*

**Cloning Primers**	**Forward Primer Sequence (5' to 3')**	**Reverse Primer Sequence (5' to 3')**
CXXC5	CGCATATACTCGAGATTACCATGGAAGCTAGCATGTCGAGCCTCGGCGGTGGCTC	CGCATGGGATCCTTTATTAGAATTCCTGAAACCACCGGAAGGCGG
EMD	CTGATCTCGAGCCATGGCTAGCGACAACTACGCAGATCTTTCG	CTGATGGGATCCTTTATTAGTCGACGAAGGGGTTGCCTTCTTCAGCCTGC
EMD ΔLEMD	TTAGTGCTAGCAGCTCGTCCGCCGCCTCCTCTTATAG	CTGATGGGATCCTTTATTAGTCGACGAAGGGGTTGCCTTCTTCAGCCTGC
FL-MAZ	CTGATGCTAGCTTCCCGGTGTTTCCTTGCACGCTGCTG	CTGATGGATCCTTTATTACCAGGGTTGGGAGGGAAGTGGCTGAGAGCT
MAZΔN	CGCATCTCGAGACGCGTAACCATGGCTAGCGTGCCCCTGAGCCTCCTGAGC	CGCATGGGATCCTCTAGATTTATTAGAATTCGCAGGTGGGCTGTGGCTGGGG
MeCP2	CTGATGATAGCGTAGCTGGGATGTTAGGGCTC	CGCATGGGATCCTTTATTAGAATTCGCTAACTCTCTCGGTCACGGG
MeCP21-400	CTGATGATAGCGTAGCTGGGATGTTAGGGCTC	CGCATGGGATCCTTTATTAGAATTCGGTGGGGTCCTCGGAGCTCTCGGG
**Tag sequences (DNA and aminoacid)**	**Nucleotide Sequence (5' to 3')**	**Amino Acid Sequence**
Flag	GATTACAAGGATGACGACGATAAG	DYKDDDDK
3xFlag	GACTACAAAGACCATGACGGTGATTATAAAGATCATGACATCGACTACAAAGACGATGACGACAAG	DYKDHDGDYKDHDIDYKDDDDK
HA	TACCCATACGATGTTCCAGATTACGCT	YPYDVPDYA
NLS (Nuclear Localization Signal)	CCCAAGAAAAAGAGGAAGGTGGGCTCACCCAAGAAAAAGAGGAAGGTGGCGGCTCA	PKKKRKVGSPKKKRKVGSS
**CXXC5 Truncation and Deletion Primers**	**Forward Primer Sequence (5' to 3')**	**Reverse Primer Sequence (5' to 3')**
3F-CXXC5	CGCATGCTAGCACCGGTTCGAGCCTCGGCGGTGGCTCC	CGCATGGGATCCTTTATTAGAATTCCTGAAACCACCGGAAGGCGG
3F-CXXC5-Δ101-149	GCAGCATGATGGGCGGAGAGCGGGCCACGGAGCTGGCAGC	GCTGCCAGCTCCGTGGCCCGCTCTCCGCCCATCATGCTGC
3F-CXXC-Δ150-199	GCCTGCTGAGCAAGGCAGAGGAAGCCCTCAATGGCCAGTC	GACTGGCCATTGAGGGCTTCCTCTGCCTTGCTCAGCAGGC
3F-CXXC-Δ200-249	TGGAGGCTGTGGCAGGTGCCGCCTCTGCCATCAGCTCCGG	CCGGAGCTGATGGCAGAGGCGGCACCTGCCACAGCCTCCA
3F-CXXC5-Δ1-100	CGCATGCTAGCACCGGTTCTGCTGACAAGGCCACTGCG	CGCATGGGATCCTTTATTAGAATTCCTGAAACCACCGGAAGGCGG
3F-CXXC5-Δ250-322	CGCATGCTAGCACCGGTTCGAGCCTCGGCGGTGGCTCC	CGCATGGGATCCTTTATTAGAATTCCAGCTCTCCCTGCATGGGGTAC
3F-CXXC5-Δ1-100 & 250-322	CGCATGCTAGCACCGGTTCTGCTGACAAGGCCACTGCG	CGCATGGGATCCTTTATTAGAATTCCAGCTCTCCCTGCATGGG
3F-CXXC	CGCATGCTAGCACCGGTGCCTCTGCCATCAGCTCCGGC	CGCATGGGATCCTTTATTAGAATTCCTGAAACCACCGGAAGGCGG
3F-CXXC-DBM	CGCATGCTAGCACCGGTGCCTCTGCCATCAGCTCCGGC	CGCATGGGATCCTTTATTAGAATTCCTGAAACCACCGGAAGGCGG
3F-CXXC5-DBM	CGCATGCTAGCACCGGTTCGAGCCTCGGCGGTGGCTCC	CGCATGGGATCCTTTATTAGAATTCCTGAAACCACCGGAAGGCGG
**Generation of DBM (AAAA-188-191)**		
1st round	CGCATATACTCGAGATTACCATGGAAGCTAGCATGTCGAGCCTCGGCGGTGGCTC	CCATGTCCGCTGCGGCAGCACCCGCCTCGCTC
2nd round	GAGCGAGGCGGGTGCTGCCGCAGCGGACATGG	CGCATGGGATCCTTTATTAGAATTCCTGAAACCACCGGAAGGCGG
Overlapping PCR (using template 1st & 2nd rounds)	CGCATATACTCGAGATTACCATGGAAGCTAGCATGTCGAGCCTCGGCGGTGGCTC	CGCATGGGATCCTTTATTAGAATTCCTGAAACCACCGGAAGGCGG
**RT-qPCR Primers**	**Forward Primer Sequence (5' to 3')**	**Reverse Primer Sequence (5' to 3')**
CXXC5	CACCAGGCATCTCTGTTGTGG	TTGTCTGCTGCTCCTGCCTTT
HDAC11	CAGCTGTGGCCCTGATCAAATC	ATCTTGGAACCCTTCTCCCTGC
NFKBIZ	ACTCAGCTGCAAAGATAAGTTCAC	ACATTATTTTGCTTGCCGCTGG
IL12A	TGCAAAGCTTCTGATGGATCC	AAAATCCGGTTCTTCAAGGGA
**ChIP Primers**	**Forward Primer Sequence (5' to 3')**	**Reverse Primer Sequence (5' to 3')**
HDAC11	CTGTGGGGGCATTTATTACG	TGCTTAAACTGGCTGCCTTT
NFKBIZ	ACGTACGCACCTTAGCCATC	AGAGAGCGAGCGATCTCCTG
IL12A	CGCTTTCATTTTGGGCCGAG	ACATCAGCTTCTCGGTGACA
Exon10 of CXXC5	TCAACCCAGGCCTCTTCATT	TGGCCAGTCTTTCGATTCCT
**Bisulfite Primers**	**Forward Primer Sequence (5' to 3')**	**Reverse Primer Sequence (5' to 3')**
HDAC11	GTTAGTTTAAGGTTATATAGTGAAGTTT	ACCAACAAATCTAAACCCTTACCTAA
NFKBIZ	GGGGGTTTAGAGGGTAGGGATGGTT	CCATAAAAATAACTCAAATTAAAC
IL12A	TATGTAAAGTGGGAGGTATTTTTTTTTTTA	CTACTACTATTCCAATCTCTCCTTAAACAA
